# A Review of Polylactic Acid (PLA) and Poly(3-hydroxybutyrate) (PHB) as Bio-Sourced Polymers for Membrane Production Applications

**DOI:** 10.3390/membranes15070210

**Published:** 2025-07-14

**Authors:** Lacrimioara Senila, Eniko Kovacs, Marin Senila

**Affiliations:** INCDO-INOE 2000, Research Institute for Analytical Instrumentation, 67 Donath Street, 400293 Cluj-Napoca, Romania; lacri.senila@icia.ro (L.S.); eniko.kovacs@icia.ro (E.K.)

**Keywords:** biodegradable polymer, biopolymer, polymer inclusion membrane, metal separation, water treatment, membrane preparation

## Abstract

In recent years, membranes have found extensive applications, primarily in wastewater purification and food packaging. However, petroleum-based membranes can be detrimental to the environment. For this reason, extensive studies are being conducted to identify environmentally friendly substitutes for the materials used in membrane composition. Among these materials, polylactic acid (PLA) and poly(3-hydroxybutyrate) (PHB) are two bio-sourced and biodegradable polymers that can be derived from lignocellulosic waste. These polymers also possess suitable characteristics, such as thermal resistance and mechanical strength, which make them potential candidates for replacing conventional plastics. This study provides an overview of recent advances in the production of PLA and PHB, with a focus on their extraction from lignocellulosic biomass, as well as the recent applications of these two biodegradable polymers as sustainable materials in membrane manufacturing. The advantages and limitations of membranes produced from these materials are also summarized. Lastly, an analysis of future trends is provided concerning new sources, production possibilities, and potential applications in water treatment (mainly for metal ions separation), gas separation, oil–water separation, medical applications, drug release control, and food packaging.

## 1. Introduction

Considering the increasing tendency of environmental contamination by inorganic and organic pollutants, the development of innovative materials and technologies for their removal is essential [1,2]. Among these, membrane technology involving organic and inorganic materials stands out for its consistent removal of a wide range of pollutants in environmental treatment. Membranes produced from organic constituents, usually polymers, are preferred due to their enhanced selectivity, efficiency, and versatility. Additionally, these membranes are cost-effective [1,3,4]. However, petroleum-based polymers are known to be non-biodegradable and are transformed into nano- and micro-plastics, which have a negative impact on the environment. Consequently, in recent years, there has been an increase in research focused on finding bio-based polymers that overcome the ecological issues produced by nano- and micro-plastics [5].

The production of bioplastics has gained increasing interest; the quantity of biodegradable polymers predicted to be produced globally in 2025 is about 1.8 million tons [6]. Among the studied materials, the biopolyesters family is a reliable substitute for petrol-based polymers [7]. Polylactic acid (PLA) and poly(3-hydroxybutyrate) (PHB) are two of the most promising biopolymers, which are biodegradable and show good mechanical performance [8].

PLA is a polyester resulting from lactic acid through enzyme fermentation of renewable biomass [9]. It is a semi-crystalline polymer with a melting temperature in the range of 130–180 °C and a glass transition temperature ranging from 50 °C to 80 °C. It has good mechanical properties, but is inferior to PVC, PVDF-HFP, or CTA [10]. Due to its good thermoplastic properties, PLA can be extruded into pellets and films, can be molded into various shapes, and can be transformed into ultrafine fibers via electrospinning [7].

PHB is produced from biomass by diverse species of bacteria and has an outstanding stereo-regularity, which provides it a remarkably high crystallinity [11]. It has a glass transition temperature of about 5 °C and a melting point in the range of 173–180 °C. PHB is a biocompatible, biodegradable, and nontoxic biopolymer, which makes it useful in various medical, environmental, and packaging applications. Due to the high cost, PHB and its blends with PLA are mainly used in medicine for drug delivery and tissue engineering [7,12,13,14,15].

To improve the mechanical strength, some authors have studied the influence of creating blends of PHB/PLA or adding different plasticizers or nanoparticles [8,16,17]. These polymers combine the advantages of being produced from renewable biomass with their biodegradability. However, more research is necessary to enhance their mechanical properties, resulting in improved performance and efficacy in membrane production [18]. By blending PLA and PHB, which have a similar chemical structure and are in the same class of biopolyesters, promising ecocompatible materials with enhanced characteristics are obtained [19]. Moreover, PLA is a less expensive material, which reduces the cost of the produced blends to a level comparable to the cost of PHB alone [20]. The use of biobased polymers in membrane compositions clearly brings environmental benefits, such as a lower carbon footprint. Okolie et al. [21] estimated that PLA manufacturing uses approximately 55% less energy and produces significantly lower CO_2_ emissions compared to the production of petroleum-based polymers. However, using biopolymers in membranes faces some challenges, mainly due to their biodegradability, which affects the robustness of the membranes and their industrial applications.

Given the limited information in the literature on the linkage between producing PLA and PHB and their use in membrane production, the aim of this review was to comprehensively present and discuss the latest findings on PLA and PHB production from renewable bio-sources and membrane production. The articles published within the last ten years were primarily analyzed to identify tendencies, and some previously published reference papers on the development of bio-sourced polymer membranes were also considered.

## 2. Methodology

To identify relevant publications related to membranes produced from PLA and PHB, as well as their applications, a search was conducted using the Clarivate database [22]. To ensure a more relevant selection, comprehensive searches were performed for the two main scopes of the current review: (1) PLA and PHB production from renewable bio-sources, and (2) membrane production from PLA and PHB, as well as their applications. Therefore, for the chapters entitled “Biodegradable and bio-sourced polymers”, “Polylactic acid (PLA) production from bio-sources”, “Poly(3-hydroxybutyrate) (PHB) production from bio-sources”, and “Characteristics of PLA and PHB”, a search was conducted for the following keywords: “biodegradable polymer” AND “polylactic acid production” OR “poly(3-hydroxybutyrate) production” AND “bio-sourced polymer” AND “characteristics of PLA” AND “characteristics of PHB”. A total of 372 English language publications from the last 10 years, categorized as “articles” and “review articles”, were found. The titles and abstracts of each publication were evaluated, and papers appropriate to the scope of this review, PLA and PHB production from renewable bio-sources, were considered. For the second part of the review, comprising the chapters “Membrane preparation from PLA and PHB” and “Applications, advantages and limitations of membranes produced from bio-sourced polymers”, a search was conducted using the following keywords: “biodegradable polymer membrane” AND “membrane preparation” AND “membrane application”. Following this evaluation, 138 articles were found to be relevant and included in the final analysis. In addition to the documents published within the last 10 years, several previously published reference works on the membrane production from bio-sourced polymers were also considered.

## 3. Biodegradable and Bio-Sourced Polymers

Biodegradable and bio-based polymers include two types of materials that have the ability to reduce environmental impact: natural biodegradable polymers and synthetic biodegradable polymers [23].

Biodegradable polymers include the following: polylactic acid (PLA), polyhydroxyalkanoate (PHA), polyhydroxybutyrate (PHB), polyhydroxyvalerate (PHV), polypropylene fumarate, polycaprolactone (PCL), polybutylene succinate, polyvinyl alcohol (PVA), cellulose acetate (CA), cellulose-based polymers, polylactide-co-glycolide, polylactide, collagen, sericin, starch, alginate, lignin, and chitosan/chitin. The bio-sources utilized are renewable biomass sources, including first-generation biomass (such as corn, sugarcane, wheat, cassava, and maize) and second-generation biomass (woody biomass and agricultural residues like straw, bagasse, and corn stover, as well as by-products and industrial waste). Lignocellulosic biomass is organic matter of biological origin and represents the most abundant source of biomass, which remains largely underutilized. It primarily consists of three polymers: cellulose, hemicellulose, and lignin, along with trace amounts of minerals, acetyl groups, and phenolic substituents. In general, the majority of agricultural lignocellulosic biomass comprises approximately 10–25% lignin, 20–35% hemicellulose, and 40–50% cellulose. Cellulose is the most abundant component of lignocellulosic biomass, while hemicelluloses—typically composed of repeated polymers of pentoses (such as xylose and arabinose) and hexoses (including mannose, glucose, and galactose)—are the second most abundant polymer. Depending on the production method, biodegradable bio-based polymers can be categorized into three groups: biopolymers produced directly from agricultural waste (such as cellulose, lignin, starch, chitin, and chitosan), biopolymers synthesized through chemical processes or polymerization from monomers derived from biomass (PLA), or biopolymers produced directly from biomass using microorganisms (PHA, PHB) [24]. Bio-sourced polymers include polymeric products obtained from renewable biological sources such as plants, algae, and microorganisms. Biodegradability depends on the environmental conditions in which they are obtained. Some polymers can belong to both categories, natural biodegradable polymers and synthetic biodegradable polymers (such as PLA, PHB, and starch blends). Currently, biodegradable polymers are used as food packaging materials. In the future, the potential for their use as agricultural engineering materials, including films and membrane separation, will be taken into consideration.

## 4. Polylactic Acid (PLA) Production from Bio-Sources

PLA is a fully biodegradable polymer derived from renewable resources. The polymeric material known as poly(lactic acid) (PLA) is synthesized from lactic acid through either chemical or enzymatic processes. The separation of lactic acid from biomass is a critical step in the process of obtaining PLA, and this subject will be addressed in a forthcoming chapter.

**Figure 1:**
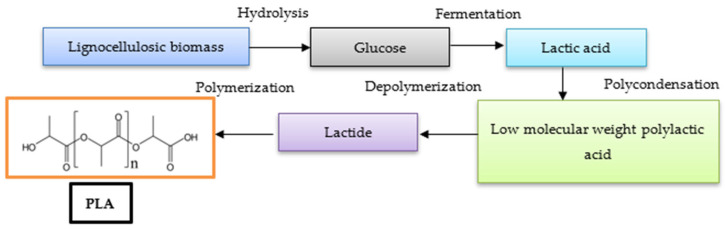
Technology for the production of PLA from lignocellulosic biomass.

Lactic acid exists in two enantiomeric forms: L(+) lactic acid and D(−) lactic acid. Pure isomers are more valuable than racemic mixtures (DL lactic acid). The synthesis of lactic acid can be achieved through biological processes, such as lactic acid fermentation, or through chemical methods. Lactic acid can be produced from renewable biomass, such as lignocellulosic materials and plants, or from other sources like sugar, oil, or fat [25]. The production of lactic acid involves various fermentation processes, multiple bacterial strains, and several purification methods. Among the purification methods, the following can be mentioned: liquid extraction, ion exchange, distillation, esterification, electrodialysis, and reverse osmosis. Lactic acid is produced on a large scale through the microbial fermentation of sugars, which are converted into cellular energy and lactate in a biological process. This production method offers several advantages, including the ability to generate optically pure D- or L-lactic acid using specific microorganisms, the utilization of inexpensive renewable raw materials, and reduced energy consumption. The optical purity and stereospecificity of lactic acid produced via fermentation depend on the microbial strain and the specificity of its lactate dehydrogenase, which influences the configuration of lactic acid as it is derived from pyruvate by the NAD-dependent stereoenzymes of L-lactate dehydrogenase (L-LDH) or D-lactate dehydrogenase (D-LDH). From a commercial perspective, lactic acid production is primarily conducted through microbial fermentation, which typically involves fermenting lactic acid from a carbohydrate source and subsequently recovering and/or purifying the resulting product. The performance of lactic acid fermentation primarily depends on the strain and the fermentation technique employed. Batch fermentation is the simplest and most commonly used industrial fermentation method. In this approach, the substrates are added to the system all at once, and fermentation continues until completion. While this closed fermentation system offers certain advantages, such as a reduced risk of contamination and the production of highly concentrated lactic acid compared to other methods, it also results in low cell concentrations. This leads to diminished nutrient levels and poor productivity, largely due to substrate or product inhibition. Achieving high cell density during fermentation is essential for maximizing the productivity of the target biomass, lactic acid, and other metabolites. The fed-batch cultivation process effectively prevents substrate inhibition and reduces the lag phase. Additionally, a well-designed substrate feeding strategy can enhance process productivity and increase the final product concentration. In comparison to batch and fed-batch techniques, continuous culture systems achieve higher lactic acid productivity and can be operated for extended periods. The repeated batch technique, which involves the reinoculation of free or immobilized cells from one fermentation batch to another, is another valuable strategy that can be applied continuously through cell recycling to attain high cell density and lactic acid productivity. The technology for the production of PLA from lignocellulosic biomass is presented in Figure 1.

**Figure 2:**
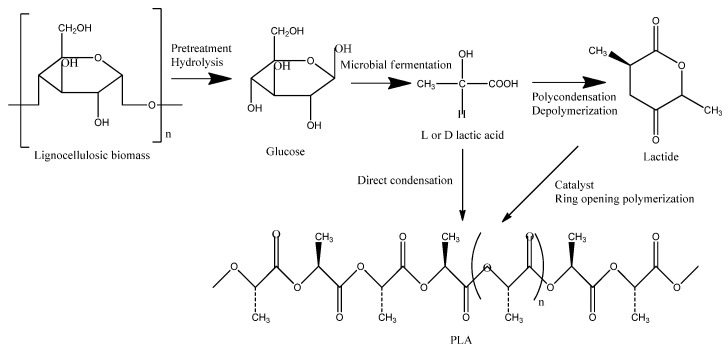
Schematic production of PLA from lignocellulosic biomass.

Microorganisms that can rapidly ferment inexpensive raw materials, requiring minimal nitrogenous nutrients, produce significant quantities of stereospecific lactic acid under high-temperature and low-pH conditions. These microorganisms generate substantial cell mass while producing minimal by-products. Lactic acid-producing microorganisms are classified into three categories: bacteria, fungi, and yeasts. Among these, filamentous fungi such as *Rhizopus oryzae* are the most well-known strains capable of producing lactic acid from glucose under aerobic conditions. Due to its amylolytic enzyme activity, *Rhizopus oryzae* can convert starch directly into L(+)-lactic acid. However, despite having lower nutrient requirements, the production rate in fungal fermentation is typically below 3 g L^−1^ h^−1^. This limitation is likely due to low reaction rates caused by mass transfer limitations and the formation of by-products, such as ethanol and fumaric acid. Large-scale production of lactic acid is predominantly achieved using lactic acid-producing bacteria, which are categorized into four main groups: lactic acid bacteria (LAB), *Corynebacterium glutamicum*, *Escherichia coli*, and *Bacillus* spp. Among these, lactic acid bacteria are the most widely exploited due to their safety, tolerance to acidic pH, and ability to generate lactic acid at very high yields and productivity. Lactic acid bacteria are a diverse group of microorganisms that play a crucial role in various fermentation processes. This group is characterized as Gram-positive, non-respiring, non-spore-forming cocci, and primarily produces lactic acid as the main product of carbohydrate fermentation. They consist of four types: *Lactobacillus*, *Streptococcus*, *Leuconostoc*, and *Pediococcus*. The new taxonomic changes included new genera such as *Alloiococcus*, *Dolosigranulum*, *Enterococcus*, *Aerococcus*, *Globicatella*, *Lactococcus*, *Tetragenococcus*, *Carnobacterium*, *Oenococcus*, *Weissella*, *Sporolactobacillus*, and *Vagococcus*. Depending on the type of lactic acid used, PLA can be divided into three main categories: poly(D-lactic acid) (PDLA), poly(L-lactic acid) (PLLA), and poly(DL-lactic acid) (PDLLA). Each type of biopolymer has different physical and chemical properties. PLLA is semi-crystalline while PDLA is amorphous. PLLA is notable due to its important properties such as crystallinity (37%), melting temperature (180 °C), and optimal glass transition (67 °C). The stability of these types of polymers is a key factor in their suitability for a range of applications, including membrane separation. The high thermal stability of both PLLA and PDLA is recommended for use in membrane separation. PDLA is optically inactive and is amorphous, which are two forms of PLA in terms of optical activity and two enantiomers derived from L (+)-lactic and D(−)-LA. The co-precipitation between PLLA and PDLA in solution creates a new polymer (called stereocomplex-type polylactide sc-PLA) which has a melting point around 230 °C, high physico-chemical properties, and will be an excellent polymer for membrane separation. PLA is produced from renewable resources, which include the use of polysaccharides from natural sources (cellulose, starch), lipids (oil and fat), and proteins (gelatine). PLA is produced by fermentation of sugars to lactic acid and polymerization of lactic acid to PLA. The polymerization of lactic acid can be achieved by three methods: (i) direct polymerization to produce a low molecular weight polymer, which is further processed to increase the molecular weight; (ii) azeotropic dehydration, which also produces a low molecular weight polymer; and (iii) ring-opening polymerization to give L-lactide, through three stages of polycondensation, depolymerization, and polymerization (Figure 2).

Generally, the catalysts used in ring-opening polymerization are metal complexes of aluminium (Al), magnesium (Mg), zinc (Zn), calcium (Ca), tin (Sn), iron (Fe), ytrium (Y), samarium (Sm), luterium (Lu), titanium (Ti) and zirconium (Zr). Among the various catalysts employed for synthesizing PLA with elevated molecular weight, stannous octoate (Sn(Oct)_2_) has emerged as the most prevalent option. Another variant is enzyme polycondensation in the presence of enzymes; a purification method of PLA is necessary [26].

**Figure 3:**
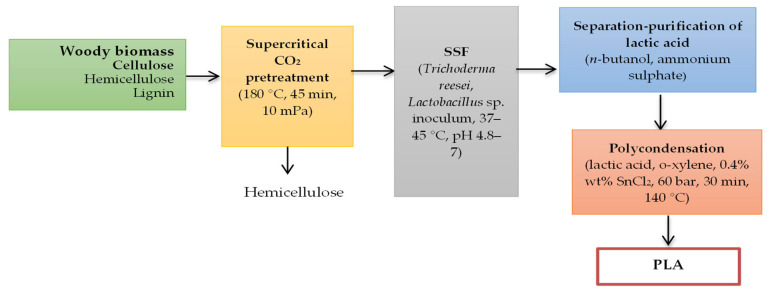
PLA production technology from lignocellulosic biomass.

Senila et al. presented a method for PLA production from lignocellulosic waste. This method involves a two-stage polymerization of lactic acid, which is obtained through pressurized hot water pretreatment and fermentation of sugars with *Lacticaseibacillus rhamnosus* [11]. The polymerization of the purified lactic acid was carried out in two stages. First, azeotropic dehydration at 140 °C for 24 h was performed in the presence of xylene and tin (II) chloride SnCl_2_ to produce lactide. Then, microwave-assisted polymerization at 140 °C for 30 min with 0.4% SnCl_2_ was used. The following technology was applied for PLA production from lignocellulosic biomass (Figure 3).

The biotechnology route corresponds to the production of PLA using microorganisms. A comprehensive review of PLA production through biotechnological methods is provided by de Albuquerque et al. In this process, lipase enzymes are employed to synthesize this polymer, which is significant for its mechanical and thermal properties. These properties make the polymer suitable for applications in the medical, pharmaceutical, and biotechnology fields [27].

Lipase B from *Candida antarctica* was used to synthesize PLA from lactic acid (LA) derived from the hydrolysate of cashew apple bagasse [28]. Recent studies have reported the use of chemically modified *Candida antarctica* lipase B (CALB), *Candida rugosa* lipase (CRL), and the cyanobacterium *Synechococcus elongatus* PCC7942 for the production of PLA [29]. The latter directly converts carbon dioxide through metabolic engineering. The heterogeneous pathway involves the introduction of D-lactic dehydrogenase, propionate CoA-transferase, and polyhydroxyalkanoate synthase into *Synechococcus elongatus* PCC7942. Several metabolic reactions take place, including promoter optimization, cetyl-CoA self-circulation, and carbon flux reactions. These processes yield an output of 15 mg of PLA/g of dry cell weight, with a molecular weight of 62.5 kDa [30]. Enzymatic polycondensation can produce polymers with a fine structure under mild conditions, without the presence of other chemicals. The polymerization methods used to produce PLA influence its stereoregularity and crystalline structure.

The preparation of membranes by using PLA requires the use of phase-separation methods, such as solvent dissolution to create a homogenous polymeric solution. The selection of an appropriate solvent is a critical step, as certain solvents can interact with the polymer and alter its chemical structure. Certain solvents interact with polymers and modify their chemical structure. In order to preserve the integrity and functionality of the polymer while ensuring its effectiveness in removing contaminants from wastewater, careful consideration of the solvent is essential. The use of toxic solvents such as dimethylformamide (DMF), dimethylacetamide, and N-methyl-2-pyrrolidone is a common practice. However, the use of green solvents such as dimethyl sulfoxide (DMSO), triethylene phosphate (TEP), and methyl lactate (ML) is considered [31].

## 5. Poly(3-hydroxybutyrate) (PHB) Production from Bio-Sources

**Figure 4:**
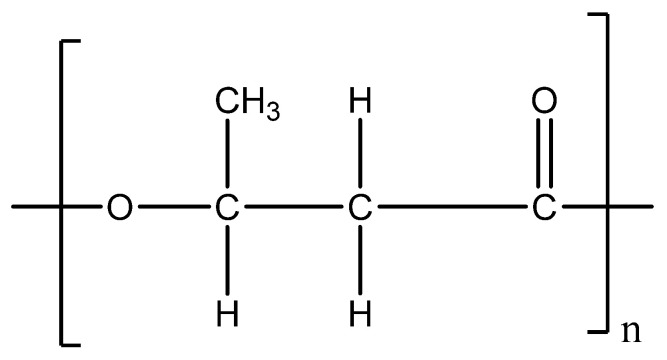
General structure of poly(3-hydroxybutyrate).

Polyhydroxyalkanoates (PHAs) are biodegradable aliphatic polyesters characterized by a structure derived from 3-hydroxyalkanoic acid (Figure 4). Polyhydroxybutyrate (PHB), a well-known bio-based polyester, was the first PHA discovered by Lemoigne in 1920 in bacteria. It is a short-chain linear homopolymer composed of repeating units of 3-hydroxyalkanoate. Numerous technologies for PHA production from different types of biomass are described in the literature. The production of PHB from renewable sources is not economically viable due to the high cost of bacteria. However, optimizing the fermentation process, enhancing bacterial cultivation techniques, and employing genetic engineering of microbial strains could significantly reduce production costs.

Short-chain polymers are similar to synthetic plastics, while medium-chain polymers are more elastic. Based on the chain length of the repeating units of the 3-hydroxyalkanoate units, there are three types of PHA: (1) short chain length (scl-PHA, with 4 or 5 carbon atoms), (2) medium chain length (mcl-PHA, with 6 or 14 carbon atoms), and (3) long chain length (lcl-PHA, with carbon atoms between 6 and 14).

PHB can be produced either synthetically or through fermentation using bacteria from various sources, including lignocellulosic biomass, rapeseed oil, sugar cane, glycerol, starch, fructose, maltose, xylose, sunflower oil, olive oil, and soybean oil. Currently, there are over 300 species of bacteria capable of producing polyhydroxyalkanoates, including the genera *Alcaligenes*, *Bacillus*, *Pseudomonas*, *Paraburkholderia sacchari,* and *Cupriavidus necator*, etc. [24].

The biomasses used for PHB production are starch (corn, potatoes, wheat), cellulose (agricultural residue, wood, plant biomass), molasses, whey, waste oil, and glycerol. PHB is a homopolymer of R-hydroxybutyrate that is biodegradable in various environments. The molecular weight ranges from 10^5^ to 10^6^ Da and can be produced by microorganisms [32]. PHB is a nontoxic, biodegradable, thermoplastic, and biocompatible material used in various applications, including agriculture, medicine, bioremediation, and membrane production. The monomers of PHB can be utilized for synthesizing pharmaceuticals, agrochemicals, and fine chemicals. Additionally, 3-hydroxybutyrate can be chemically modified for use in diverse applications.

Lin and Ng reported on a method for producing PHB from molasses using *Cupriavidus necator* H16 in a fed-batch fermentation process [33]. This method employed a carbon-to-nitrogen (C/N) ratio of 20:1 and phosphorus enrichment under nitrogen-limiting conditions. In contrast, Kranert et al. described the production of PHB using *Cupriavidus necator* with fructose and propionic acid as carbon sources [34]. Heterotrophic bacteria, such as *Cupriavidus* sp., particularly *Cupriavidus necator*, have been extensively utilized for synthesizing PHB due to their high capacity to accumulate PHB under nutrient-limited conditions, especially when an excess carbon source is available [35]. Russo et al. reported the production of PHB from cardoon stalks using *Cupriavidus necator*, yielding the following results: dry weight (DW) of 7.58 g/L, 77% PHB content, PHB production (PHBp) of 5.84 g/L, and a yield of PHB (Y_PHB_) of 0.26 g/g [36]. According to Senila et al., PHB was produced from lignocellulosic waste through the following steps: microwave irradiation, ammonia delignification, and enzymatic hydrolysis using *Bacillus megaterium* ATCC [11]. Li et al. reported the concurrent production of bioethanol and PHB by *Zymomonas mobilis*. The integration of genetic and process engineering methodologies resulted in the development of a novel microorganism, which was achieved through the overexpression of the *pha*CAB gene using a strong promoter, thereby augmenting its copy number [37].

**Figure 5:**
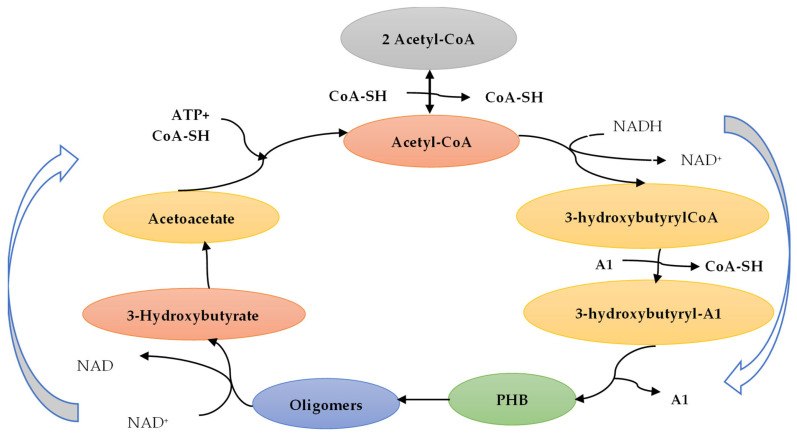
Production of PHB and degradation process. Reproduced from [38], *Polymers*, 2020.

Cyanobacteria, often referred to as blue-green algae, are photosynthetic prokaryotes capable of synthesizing polyhydroxybutyrate (PHB) as a carbon and energy storage material in response to stress conditions, such as nutrient limitation. This bacterium requires a supplementary carbon source. Cyanobacteria can produce polyhydroxybutyrate (PHB) by utilizing carbon dioxide (CO_2_) as both an energy and carbon source. Notable species include *Aulosira fertilissima*, *Synechococcus* sp., *Calothrix scytonemicola* TISTR 8095, and *Nostoc muscorum*. In general, the PHB yield produced by cyanobacteria is significantly lower than that of *Cupriavidus necator* and is produced only at the lab scale. Figure 5 presents the production of PHB by bacterial fermentation using the carbon metabolite from acetyl-CoA.

## 6. Characteristics of PLA and PHB

PLA and PHB are the most widely studied polymers derived from renewable resources, being potential alternatives to plastics based on petroleum. PLA is a biodegradable polymer obtained by ring-opening polymerization of lactide or by direct polycondensation of lactic acid. The mass of PLA acid depends on the purity of the lactic acid utilized and its designation as L(+) or D(−) [39]. PHB is produced through microbial biosynthesis as a reaction to excessive carbon and is a biodegradable polymer that belongs to the polyhydroxyalkanoate family. It has a methyl functional group (CH_3_) and an ester linkage group (−COOR) [38]. Understanding the properties of these two biobased polymers is critical to the optimization of their use in a variety of applications. A comparison of the key characteristics of PLA and PHB is presented in Table 1.

As shown in Table 1, a clear distinction can be observed in the physico-chemical and mechanical, thermal, barrier, and biodegradability properties exhibited by the two analyzed polymers, making them suitable for different applications. Compared to other polymers, PLA offers several advantages: it is eco-friendly, as it comes from renewable sources; it is biocompatible, with no toxic or carcinogenic effects; and it has superior thermal processability. It can be processed through injection molding, film extrusion, blow molding, fiber spinning, and film forming, which saves energy. PLA has a stereochemical structure, and depending on the type (PLA, PLLA, PDLLA), its physical properties differ [41]. PLA can be semicrystalline or amorphous. Semicrystalline PLA has regular repeated units, giving it higher tensile strength and modulus compared to amorphous PLA. No polymer is completely crystalline, as they usually contain both amorphous and semicrystalline regions. Crystallinity plays a significant role in the physical properties of polymers, such as their thermal and mechanical effects, as well as their biodegradability. Additionally, crystallinity influences hardness, modulus, tensile strength, stiffness, and melting points. The glass transition temperature (Tg) and the melting temperature (Tm) are two important parameters that influence polymer flexibility and processability.

In the case of amorphous polymers, flexibility is reduced when polymers are cooled below their Tg. The Tm and the degree of crystallinity depend on the molar mass and purity of the polymers. PLA is insoluble in water, alcohol, and linear hydrocarbons. PLA is soluble in dioxane, acetone, acetonitrile, chloroform, dichloroacetic acid, and methylene chloride. PLA can be dissolved in toluene, acetone, ethyl benzene, and tetrahydrofuran when heated to their boiling temperatures. The solubility of PLA is influenced by the crystallinity of the polymer. PLLA is crystalline and cannot be dissolved in acetone, ethyl acetate, or tetrahydrofuran.

In order to enhance the mechanical and thermal properties of PLA, various materials are used for blending, such as plasticizers and nucleation agents [25,42,45]. The molecular weight of a polymer significantly influences its degradation, mechanical strength, and solubility. A low molecular weight leads to rapid degradation, while a high molecular weight increases the degradation rate.

Although PLA and PHB have roughly similar chemical structures, they are not miscible. PLA is the most widely used polymer in various applications. The chemical properties of PLA are characterized by its ability to undergo hydrolysis reactions. PLA is characterized by its ability to be broken down through a hydrolysis reaction. The chemical properties of PLA are significantly influenced by the stereochemistry of its monomers. The pH level affects the hydrolysis of ester bonds and is favored at low pH values [41].

The mechanical properties of various types of PLA differ concerning the following characteristics: tensile strength, elongation at break, modulus of elasticity, yield strength, flexural strength, unnotched Izod impact, Rockwell hardness, heat deflection temperature, and Vicat penetration. Higher mechanical properties are associated with semicrystalline PLA. PLA is hydrophobic and has a low functionalization capacity; however, applying surface modifications can enhance its hydrophilicity. There are numerous chemical and physical methods used to improve the hydrophilicity of PLA. One simple approach is to blend it with hydrophilic polymers, such as starch and citrate plasticizers. The morphology of the fibers plays a significant role in determining hydrophilicity. The introduction of specific functional groups can modify hydrophilicity at the molecular level. Other methods for surface modification include plasma treatment, UV irradiation, grafting, and blending with various polymers and additives [63].

PHB is produced by bacteria, which is a limiting factor for large-scale production due to the costs associated with the extraction process. It is a homopolymer of R-3-hydroxybutyrate. The PHB monomer is a chiral molecule and can be used as a chiral precursor. It is water-insoluble, and it is characterized by good biodegradability, biocompatibility, and oxygen permeability [32]. However, its high brittleness and stiffness, together with its low thermal stability and limited chemical resistance (which can be affected by alkalis and acids in chlorinated solvents), have been identified as drawbacks in its properties [46,64]. To address the limitations of PHB, the polymerization process can be modified by incorporating monomers, such as 3-hydroxyvalerate and 3-hydroxyhexanoate, and by blending with plasticizers such as starch, chitosan, polycaprolactone, polyethylene glycol, and PLA. This approach has the potential to enhance the performance of the polymer [43]. PHB has many applications in the biomedical, catalysis, biosensors, and adsorbents fields. In this context, various processes are employed to decrease crystallinity, lower the melting point, and enhance mechanical properties. D’Amico et al. investigated PLA/PHB blends produced by melting in various ratios to assess their crystallinity, miscibility, mechanical properties, and permeability. The study demonstrated that these fully biodegradable blends exhibited increased crystallinity, immiscibility (evidenced by distinct glass transition and melting temperatures), and improved mechanical properties, such as greater elongation at break and enhanced polymer chain mobility. Notably, the PHB/PLA (30/70) blend emerged as a promising alternative to conventional, non-biodegradable plastics [8]. The same results were confirmed by Zhuikov et al., demonstrating a high enzymatic degradation of the biopolymer (50:50 PHB/PLA) in comparison with the pure polymers (PLA and PHB) [65].

PHB contains a methyl group and exhibits hydrophobic characteristics. The regularity of the polymerized structure influences its crystallinity. Isotactic PHB features a chiral carbon with an absolute configuration and is produced through bacterial fermentation, whereas syndiotactic PHB is synthesized via a synthetic route from monomers with either R or S configurations. Isotactic PHB has a more regular structure, resulting in higher crystallinity [66].

PHB has a hydrophobic character due to the presence of methyl and alkyl groups in its structure. PHB has high crystallinity, which is associated with strong intermolecular forces that may improve its mechanical properties, especially its tensile strength. PHB is relatively stable at neutral pH. However, it can be enzymatically degraded at elevated temperatures, specifically at a pH of 7 and a temperature of 55 °C. PHB is susceptible to degradation under both alkaline and acidic conditions. In alkaline environments, this degradation results in the formation of 3-hydroxybutyric acid, crotonic acid, or both [62]. Hydrophilicity could be enhanced by blending with other hydrophilic polymers or through chemical and physical modifications. Studies have demonstrated that blending PHB with synthetic or natural polymers decreases crystallinity, increases surface hydrophilicity, reduces brittleness, and enhances degradation. The degradation rate is influenced by the polymer’s hydrophobic-hydrophilic balance. Blending induces various physico-chemical changes. By blending PHB with chitosan, lignin, polyethyleneoxide (PEO), cellulose, and other materials, hydrogen bonding forms between the carbonyl groups of the PHB and the amide or hydroxyl/ether groups of the additives. This interaction enhances hydrophilicity, reduces brittleness, accelerates degradation, and decreases the water contact angle [67]. Akdoğanc et al. (2024) reported a reduction in the water contact angle of PHB from 98° to 57° and an increase in surface free energy by applying plasma surface treatment [68].

A critical evaluation of the characteristics of PLA and PHB is presented in Table 2, based on information from the literature [48,69].

Overall, PLA is more suitable for commercial applications (packaging, 3D printing) due to its higher tensile strength and higher glass transition temperature. PHB, on the other hand, is more appropriate for specialized applications (biomedical, food, films) in which good thermal resistance, fast biodegradation, and good barrier properties are needed.

## 7. Membrane Preparation from PLA and PHB

A membrane is defined as a semipermeable material that can be used to separate different substances based on their properties. According to their morphology, membranes can be classified as porous or non-porous. Depending on their constituents, membranes can also be organic or inorganic. Because these are easy to process and have higher selectivity, organic membranes are chosen for both laboratory scale and industrial applications [70]. The use of bio-based polymers to produce organic membranes makes them biodegradable by enzymatic or non-enzymatic hydrolysis. Because membranes are converted to compost following hydrolysis, the introduction of harmful substances in biodegradable polymers should be avoided [71]. Originally, biodegradable polymers were used in the packaging industry. With the development of new types of biopolymers with improved characteristics, mainly regarding their mechanical and thermal resistance, the areas of membranes produced from biopolymers were extended in domains involving membrane separation, such as reverse osmosis, microfiltration, ultrafiltration, dialysis, electrodialysis, and gas separation [70]. To enhance the mechanical stability, fouling resistance, and hydrophilicity of membranes made from biodegradable polymers, several procedures such as self-assembly, blending, surface grafting, or coating have been applied [72,73].

### 7.1. Preparation Methods of Bio-Based Polymer Membranes

**Figure 6:**
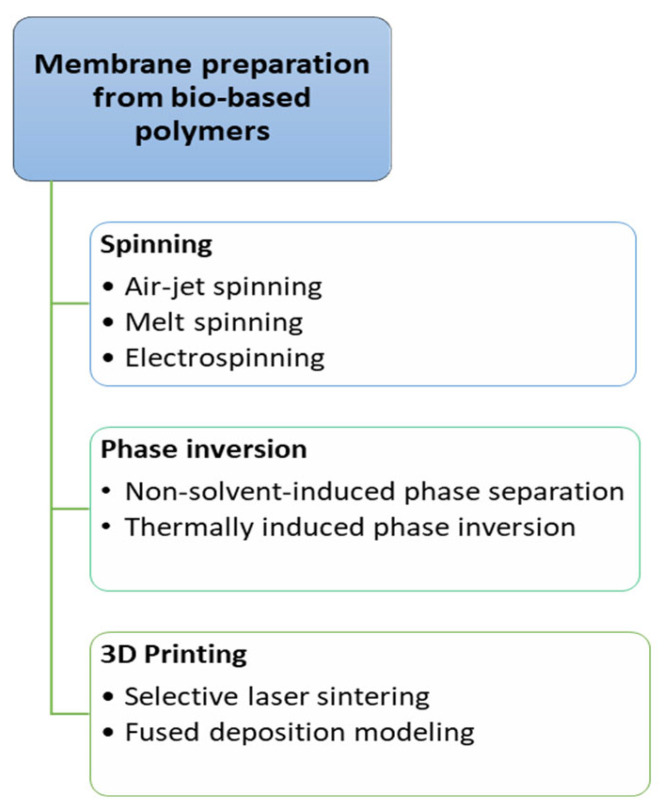
Principal preparation methods of bio-based polymer membranes.

According to the literature, there are several methods of membrane preparation from polymers [70]. The main methods used to prepare membranes from bio-based polymers are presented in Figure 6.

The expansion of membrane filtration technology employing polymers holds great potential for efficient water treatment. The produced membranes can be used for the removal of metal ions or oils from wastewater [1]. Thus, there has been an increasing interest in identifying cost-effective and environmentally friendly methods for the production of polymeric membranes. As shown in Figure 6, there are three main methodologies used to prepare membranes from bio-based polymers: (1) spinning, (2) phase inversion, and (3) 3D printing.

#### 7.1.1. Spinning

**Air-jet spinning**

In this preparation method, the polymer is dissolved in appropriate solvents and is squeezed out through a nozzle to produce thin fibers. The polymer fiber is solidified in synchronization with the solvent evaporation. This cost-effective method was applied to prepare the PLA membrane by dissolving PLA in chloroform and ethanol (ratio 3:1), resulting in 7% and 10% PLA solutions. The solution was extruded at a pressure of 30 psi and a flow rate of 0.27 mL/min [74]. The air-jet spinning method requires a specific spinning system nozzle, such as a commercial airbrush, as well as a surface for gathering polymer fibers. Compressed gas is introduced into the polymer solution, which is then ejected to create the fiber. This technique has several advantages: it is easy to use, fast, inexpensive, and does not require high voltage [74]. This method was also applied to produce nanofiber scaffolds of nanohydroxyapatite/PLA [75].

**Melt spinning**

This fabrication method was developed in order to obtain nanofibers by means of the melt extrusion of immiscible blends of bio-based polymers and thermoplastic polymers. Tran et al. reported the use of this technique for the preparation of thermoplastic nanofibrillary structures from poly(vinyl alcohol) and PLA blends [76]. In another study, this method was used to produce a fibrous composite membrane from PLA as matrix and polyvinylidene fluoride (PVDF) micro and nanofibers [77]. Zhu et al. (2023) produced an antibacterial PLA filter using the melt-blown spinning method [78]. To improve the spinnability of PLA, the authors used an irradiation technique performed through a γ-irradiator. The obtained PLA material was used for air purification tests and to filter *Escherichia coli* and *Staphylococcus aureus* bacteria. The tests indicated that PLA is a promising candidate for the large-scale production of antibacterial filters. Recently, Guo et al. (2025) used melt blowing, followed by an electrospinning process to produce nonwoven fabrics [79]. Materials based on hydrophobic PLA offer efficient air filtration and are degradable, making them an eco-friendly alternative to classical filtration materials.

**Electrospinning**

**Figure 7:**
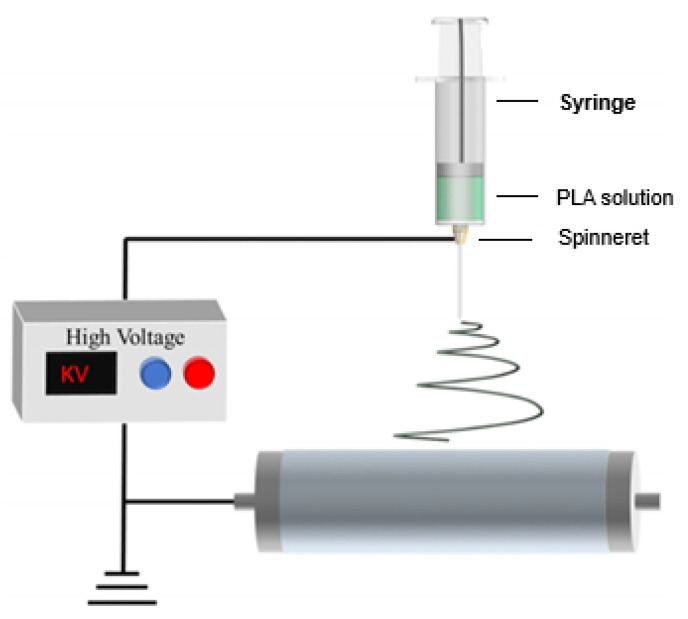
Preparing a membrane by electrospinning technology. Reproduced from [81], *Polymers*, 2024.

In electrospinning, an electrical voltage is applied to a solution of a polymer dissolved in a solvent. When the liquid droplet becomes electrified, a jet is generated, which is then stretched and elongated to produce fibers. The resulting membrane is characterized by a high hydrophobicity [80]. The process of membrane preparation by the electrospinning technology is presented in Figure 7 [81].

The characteristics of PLA nanofibers can be manipulated by changing some electrospinning parameters, such as spinneret diameter, applied voltage, and distance from the spinneret to the receiving substrate [59]. Zhang et al. [82] combined surface functionalization with emulsion electrospinning multistructural PLA-based nanofiber membranes with application in drug delivery. High-efficiency air filters were produced from PLA for capturing ultrafine particulate matter PM_0.3_ from air using the electrospinning method [83]. The advantages of the produced filters are their excellent filtration efficiency and their biodegradability, which makes them environmentally friendly.

#### 7.1.2. Phase Inversion

**Non-solvent-induced phase separation (NIPS)**

**Figure 8:**
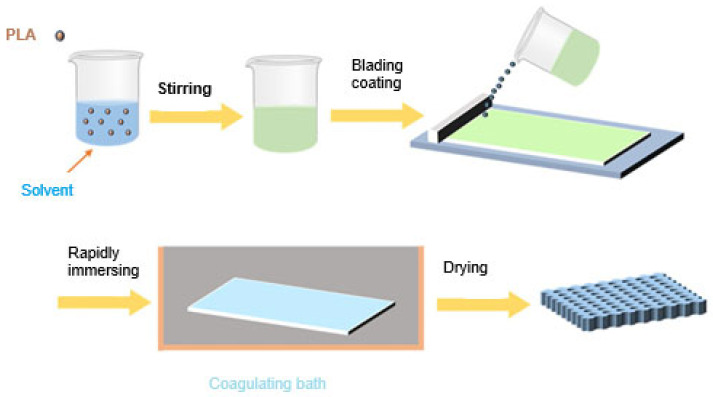
Schematic representation of the PLA membrane preparation by the NIPS method. Reproduced from [81], *Polymers*, 2024.

This method is typically used for producing porous membranes used for filtration [84]. It is based on the development of two phases: the first phase contains a polymeric solution which is in charge for the growth of the membrane matrix, and the second phase contains a very low amount of the polymer, which is washed out throughout the membrane formation causing the growth of pores when the membrane is solidified. Figure 8 illustrates the steps involved in the production of PLA membranes using the NIPS process [81].

One of the shortcomings of NIPS is that it requires strong solvents, which result in a high volume of solvent-polluted wastewater [70].

**Thermally induced phase inversion (TIPS)**

**Figure 9:**
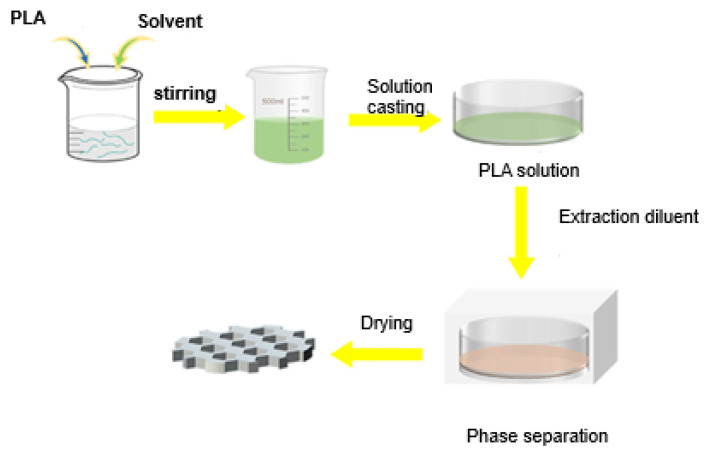
Schematic representation of the PLA membrane preparation by the TIPS method. Reproduced from [81], *Polymers*, 2024.

Thermally induced phase separation (TIPS) is similar to the NIPS method, but differs in the use of semi-crystalline polymers. In the TIPS method, a solvent is used that works at a high temperature, close to the polymer’s melting point. Potential solvents for the TIPS method include triethylene glycol diacetate (TEGDA), acetyl tributyl citrate (ATBC), and sulfolane [70]. The polymer dissolved in the solvent is cooled for phase separation. The membranes obtained by this method display a porous and highly symmetrical structure. The main drawback of both NIPS and TIPS methods is that highly toxic solvents are commonly used to produce membranes [85]. Figure 9 presents the steps of the TIPS process to produce a PLA membrane [81].

#### 7.1.3. 3D Printing

Three-dimensional (3D) printing has undergone an outstanding evolution and an exponentially growing application [86]. Three-dimensional printing can be used to produce membranes of different forms, allowing the production of tailored pieces for various purposes. Biodegradable polymers can be used for medical applications [87]. In case of PLA, brittleness and its poor melting property may cause problems in extrusion foaming and poor product performance. For this reason, improving the rheological characteristics of PLA is necessary to enhance the foamability and morphology of the material before printing [88]. Selective laser sintering (SLS) and fused deposition modeling (FDM) are among the promising 3D printing procedures.

**Selective laser sintering (SLS)**

In this method, a powdered polymer is spread across a surface and then subjected to sintering using a laser to create the wanted structure. This process is repeated in order to produce layer by layer the membrane [70,89]. Using this method, less porous materials can be obtained that are stable from a mechanical point of view. SLS was applied to produce 3D nanocomposite scaffolds for bone tissue from a carbonated hydroxyapatite (CHAp)/poly(L-lactic acid) (PLLA) material [90].

**Fused deposition modeling (FDM)**

In the fused deposition modeling method (FDM), the membrane is produced by heating the polymer above the glass transition temperature. The membrane can then be printed through an extrusion nozzle. This method is considered cost-effective and does not require toxic solvents to produce the membrane [86]. Rosenzweig et al. produced a membrane from PLA and acrylonitrile butadiene styrene (ABS) using the FDM method [91]. This membrane offers improved mechanical stability and compressibility for application in cartilage and intervertebral disc tissue regeneration. The FDM method was also used for 3D printing of PLA for biomedical applications. To improve membrane properties, cyclic peptides were conjugated to the surface of gold nanoparticles for bone remodeling [92].

A critical evaluation of the possible advantages and disadvantages of the preparation methods is presented in Table 3.

### 7.2. Materials Used for Membrane Preparation

#### 7.2.1. Membrane Preparation from PLA

Since PLA is biodegradable, easy to produce, and can be obtained from renewable bioresources, it is the most promising and competitive material for membrane production. Lactic acid is the precursor of PLA and can be conventionally obtained from biomass. Despite its many advantages, PLA has several drawbacks like brittleness at room temperature, fragility, and a relatively low degradation rate [93].

A homopolymer is obtained when PLA contains either optically pure D-lactic acid or L-lactic acid. However, PLA can be produced from both lactic acid enantiomers, resulting in a heteropolymer. The proportions of lactic acid enantiomers in the PLA chain influence some characteristics of PLA like melting and glass transition temperatures and the grade of crystallization. While PLA homopolymers are semicrystalline, PLA heteropolymers are amorphous [94].

**Figure 10:**
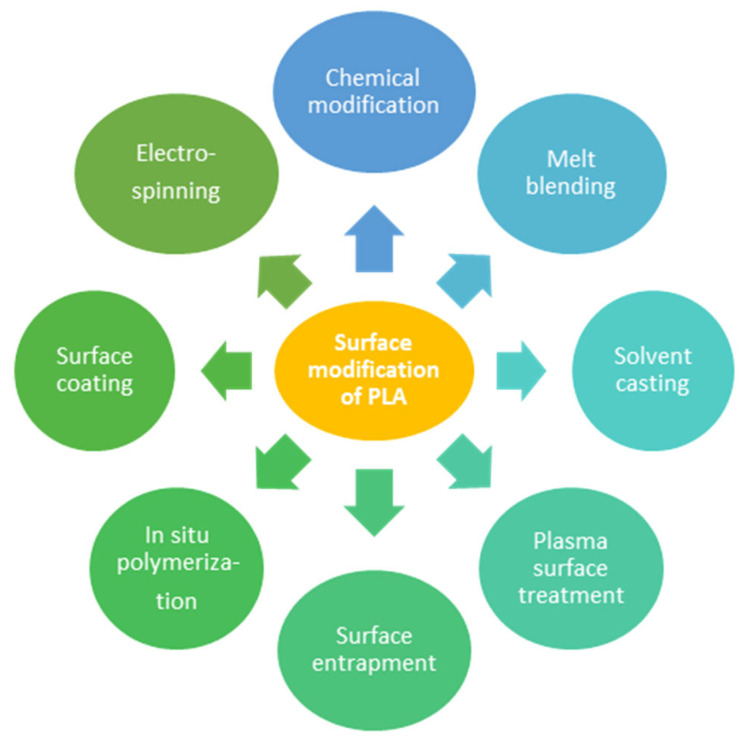
Surface modification of PLA used for membrane preparation.

To improve PLA’s performance, a significant research effort was carried out on finding appropriate copolymers, plasticizers, flexible polymers, rubbers, or rigid fillers [16]. Additionally, surface modification of PLA can improve its properties and expand its array of applications. For instance, several methods can be used to modify the surface of PLA to enhance its bioactive features. The main methods of surface modification used in the enhancement of PLA properties are presented in Figure 10 [95].

**Chemical modification.** The chemical modification of PLA involves the addition of other molecules to the PLA surface to improve its characteristics for specific applications [95]. The most important example of the chemical modification of the PLA surface is the formation of hydroxyl and carboxylic acids and groups by alkaline hydrolysis [96,97]. In other studies, carboxyl and amino groups were introduced to modify the side chain of PLA, with a positive effect on the polymer’s biodegradability conservation [98].

**Melt blending.** In the melt blending procedure, PLA is mixed with bioactive compounds throughout the melt processing phase at high temperatures exceeding its melting point, and under high pressure [99]. Potential problems that may occur during this process include thermal degradation of PLA and the formation of LA monomers. Additionally, this process requires high energy consumption [100]. PLA can also be mixed with other materials, such as additives, nanoparticles, and natural ingredients [101,102]. Melt blending was also used by Backes et al. [103] to produce biocomposites encompassing PLA and tricalcium phosphate, with application in 3D printing.

**Solvent casting.** In the solvent casting process, PLA and a bioactive compound are dissolved in an appropriate solvent and then mixed to obtain a homogenous dispersion. The resulting blend is then cast onto a substrate or into a mold. Once the solvent evaporates, a PLA composite with the bioactive compound forms [95]. This process was used, for example, to produce biocomposites from PLA mixed with piper betle fiber, with improved tensile properties [104]. Films with antioxidant properties were produced using the solvent casting method from PLA, integrating carotenoid extracts [105]. Although solvent casting is a simple process, it is time-consuming and energy-intensive, thus limiting its scalability [106].

**Plasma surface treatment.** Plasma surface treatment involves exposing PLA to a plasma formed from an ionized gas containing primarily ions, electrons, and free radicals. The interaction between the material surface and the plasma breaks molecular chains, generating other functional groups [107]. Using this method, functional groups such as hydroxyl and carboxyl can be added to the PLA surface, increasing its hydrophilicity [108]. Recently, this technique has been used to add compounds with biomedical applications [109,110] or antibacterial agents [111] to the PLA surface. The limitations of the plasma surface treatment technique are the loss of material effectiveness due to surface rearrangement over time, or increased polymer degradation when high plasma power and a long treatment period are applied.

**Surface entrapment.** This method integrates molecules that do not adhere to PLA without the need for reactive side chain groups on its surface. This method can modify the PLA surface by introducing a second polymer, such as poly(L-lysine), poly(ethylene glycol) [112], or poly(DL-lactide) [113]. More recently, Wang et al. used the surface entrapment technique to modify the surface of PLA by introducing chitosan [114]. Due to its advantages, the entrapment method can be further developed for use in biomedical applications.

**In situ polymerization.** This method incorporates a bioactive compound into the PLA throughout the polymerization process. The advantage of this process is that it provides good control over the integration of the bioactive compound within the PLA matrix [95]. This method was used to create a bioactive PLA incorporating polyethylene glycol and lisinopril [115]. Additionally, this process can be used to incorporate inorganic compounds, such as titanium oxide [116], demonstrating its versatility for various applications.

**Surface coating.** In the surface coating method, a modifying constituent is deployed on the surface of the polymer. This method was widely studied for the modification of PLA for use as a drug carrier and in regenerative medicine. The obtained materials can usually be tailored to match specific anatomical necessities, being suitable for prototyping with 3D printing [117]. Using this method, Nazeer et al. [118] added hydroxyapatite and chitosan to the PLA surface, while other authors added poly(vinyl alcohol) nanohydroxyapatite nanofibers to the PLA surface [119].

**Electrospinning.** The electrospinning process transforms PLA combined with surface modifiers into nanofibers with an increased surface area. In this method, a PLA solution and modificatory substance are electrostatically spun into nanofibers. Using electrospinning, Imani et al. [120] integrated polypyrrole grafted gelatine into PLA nanofibers. In another study, bioactive glass and magnesium oxide were incorporated into electrospun PLA fibers [121].

In general, surface modification of PLA greatly expands its application domain, mainly toward biomedical applications, drug delivery, and 3D printing. However, in some cases, increased costs may limit the development of this technology on an industrial scale.

#### 7.2.2. Membrane Preparation from PLA–PHB Blends

The use of PLA and PHB has remarkable advantages, but also some disadvantages. For instance, PHB is mechanically fragile and its melting temperature is very close to its degradation temperature, whereas PLA has a high glass transition temperature, which determines the brittleness of the obtained products [8]. To eliminate these drawbacks, blends of PLA/PHB were formulated. These blends also include plasticizers such as polyethylene glycol or limonene, or other copolymers [8,16,122,123]. D’Amico et al. prepared biodegradable polymeric blends based on PHB and PLA by melt mixing [8]. The authors used trybutyrin as a bio-based plasticizer. Tensile tests of the obtained blends showed that the elongation at break increased proportionally with the increase in PLA content. The obtained blends presented improved characteristics that allow us to extend the applications of PLA and PHB. Arrieta et al. created PLA/PHB electrospun materials using the electrospinning technique [122]. Different ratios (0:100, 25:75, 50:50, 75:25, and 100:0) of PLA/PHB were used to produce electrospun fibers. Bartczak et al. prepared blends of PHB/PLA containing up to 20% PHB [16]. The authors reported that PLA and PHB were partially miscible. The tensile impact resistance grows from around 50 kJ/m^2^ of films made only from PLA to about 120 kJ/m^2^ in a blend with 80:20 PLA/PHB. Jašek et al. developed a depolymerization procedure of PLA and PHB using bio-based solvents to obtain alkyl esters which were then modified into polymerizable molecules by methacrylation with methacrylic anhydride [124].

The characterization of polymers and the obtained membranes has a critical role in understanding their morphology, structure, mechanical properties, and thermal stability. Among the commonly used techniques, Fourier-Transform Infrared Spectroscopy (FTIR) and Raman spectroscopy offer valuable information regarding the chemical composition and functional groups. Scanning Electron Microscopy (SEM), and X-ray diffraction (XRD) provide information about the crystalline structure and degree of crystallinity of membranes. Thermal characterization, consisting of thermogravimetric analysis (TGA) and Differential Scanning Calorimetry (DSC), offers valuable information regarding the thermal behavior of membranes. Mechanical properties refer mainly to tensile characteristics. The static water contact angle measurement characterizes the membrane surface wettability, whereas the disintegration under composting conditions test characterizes the membrane degradability.

## 8. Applications, Advantages, and Limitations of Membranes Produced from Bio-Sourced Polymers

**Figure 11:**
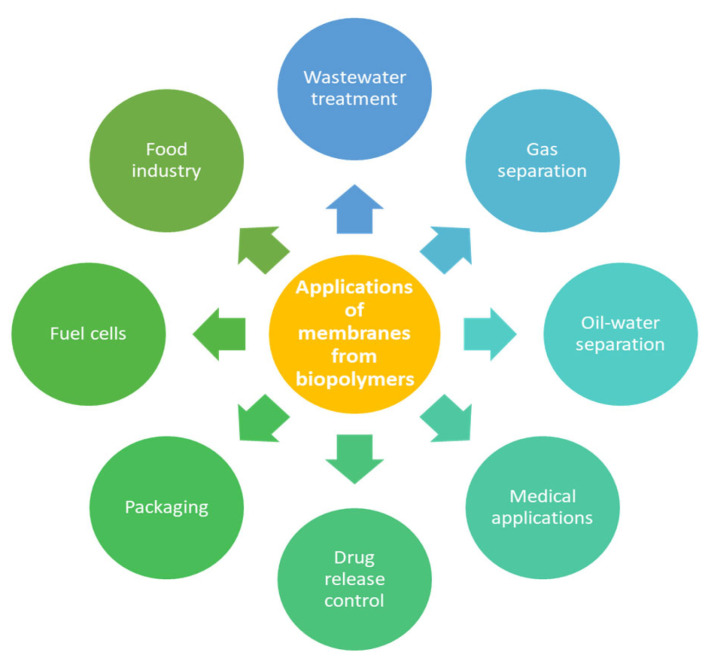
Main applications of membranes produced from bio-sourced polymers.

Membranes produced from bio-sourced polymers have been used in diverse applications. Figure 11 displays the main applications of these membranes.

**Water treatment**

Adsorption membranes are used for the removal of different contaminants from wastewater through chemical or physical interactions on their surface [71,125]. A PLA-based membrane containing hydroxyapatite (HAp) nanoparticles as the adsorbent was developed and used to remove cationic and anionic metals [126]. This type of adsorptive membrane exhibited good mechanical strength, high water flux (1100 L/m^2^·h), high porosity (78%), and very good removal efficiency for Pb and As. Porous PLA/chitosan nanofibers were tested for Cu ions absorption [127]. In another study, PLA nanofibers were modified with polydopamine to increase the chitosan grafting [128]. Another PLA adsorptive membrane containing TiO_2_ as a photocatalyst was produced for methylene blue removal [129].

One recent application of PLA membranes is the separation of metal ions for water treatment. Recently, Modolon et al. [130] prepared PLA-based membranes by the electrospinning process in combination with chitosan for the adsorption of Cd^2+^, Cu^2+^, Mn^2+^, Ni^2+^, and Zn^2+^ ions from aqueous solutions. The incorporation of chitosan into the PLA membrane reduced porosity, improving tensile strength and elasticity. The obtained membranes exhibited removal efficiency values ranging from 5 to 17%, and an affinity toward different anions in the order Cd^2+^ ≅ Cu^2+^ ≅ Zn^2+^ > Mn^2+^ ≅ Ni^2+^. Dual-layer membranes were produced using PLA/poly(butylene succinate) (PBS) and cellulose nanowhiskers (CNWs) as fillers for the removal of heavy metal ions from water [131]. The membrane exhibited high removal efficiency, especially for Co^2+^ and Ni^2+^ (83% and 84%, respectively). Prazeres Mazur et al. [132] prepared biomembranes containing PLA, PBS, or their composites and blends by the electrospinning process, with adsorptive materials for Cd^2+^, Cu^2+^, Cr^6+^_,_ Mn^2+^, Ni^2+^, and Zn^2+^ ion removal from the aqueous solution. The membrane characterization confirmed that the addition of PLA and nanoclay resulted in the development of a resistant material, with a higher porosity and specific surface area than when using neat PBS. The adsorption assays showed selectivity series of membranes as follows: Cd^2+^ ≅ Zn^2+^ > Cu^2+^ > Ni^2+^ > Mn^2+^. Mokoena and Mofokeng [133] tested a membrane prepared from PLA/poly(3-hydroxybutyrate-co-3-hydroxyvalerate) (PHBV)/GO composites for the removal of Pb^2+^ in water. A membrane with 1% GO loading provided the highest Pb^2+^ adsorption efficiency (81.3% to 86.5%). In a recent study, Nigiz et al. [134] prepared PLA-based membranes loaded with a zirconium metal–organic framework (MOF) used in membrane desalination and pervaporation systems for boron separation. The authors reported that both methods provided 99.9% boron removal. Shokri et al. [126] prepared adsorptive membranes from PLA/hydroxyapatite (HAp) component, with a variable HAp content. A membrane containing 2.5 wt% HAp exhibited high hydrophilicity and porosity, and satisfactory mechanical strength. Batch experiments conducted on contaminated water showed that the membrane can remove up to 97% Pb and 82% As from solution.

Polymer inclusion membranes (PIMs) are a new type of membrane used for metal separation. Bożejewicz et al. [135] prepared polymer membranes using different polymers such as PVC, CTA, PLA, plasticizers, and carriers for the removal of Cd^2+^ ions from aqueous systems. The membranes exhibited good adsorptive properties, providing a fast Cd^2+^ removal. The membrane prepared using PLA was considered advantageous since this polymer is biodegradable. Hammadi et al. [136] used the evaporation casting method to prepare a new PIM containing PLA mixed with polybutylene adipate terephthalate (PBAT) as base polymers and Aliquat 336 as the ion carrier and either Cloisite 30B or graphene oxide (GO) as nanofillers. The resulting materials, particularly those filled with GO, showed high extraction efficiency of Cr(VI).

In other studies, the bio-based membranes were used for wastewater treatment, for the removal of metal ions and other contaminants. Matei et al. [137] prepared two types of membranes by the electrospinning process: one containing PLA and PLA/PHB, and the other using chitosan and activated coal to coat the PLA/PHB membrane. The membranes showed high efficiency in removing suspended solids and Pb^2+^ ions from wastewater. Cairone et al. [138] prepared negatively charged and positively charged PLA nanocomposite mixed matrix membranes and used them for the removal of heavy metals, nutrients, and organic pollutants from municipal wastewater. In general, the system achieved over 80% removal efficiency for chemical oxygen demand, nitrate, phosphate, ammonium, zinc, nickel, and copper.

**Figure 12:**
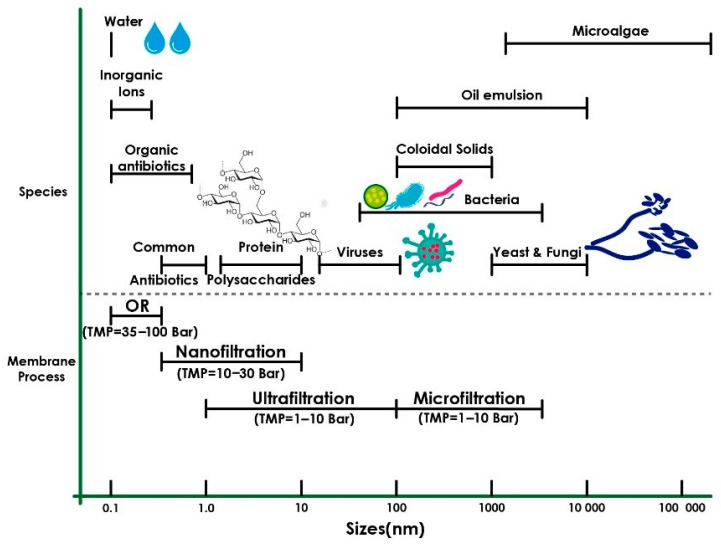
The influence of membrane pore size on their applications. Reproduced from [139], *Membranes*, 2023.

The pore size of membranes strongly influences their ability to remove different contaminants from water. The processes in which membranes are used according to their porosity are microfiltration (MF), ultrafiltration (UF), nanofiltration (NF), and reverse osmosis (RO). A classification of these processes and the filtered components is shown in Figure 12 [139].

**Gas separation**

Lehermeier et al. used a PLA-based polymer as a gas separation membrane for the first time, with application in hydrogen purification [140]. More recently, Akbarzadeh et al. produced a thiazole-based polymeric membrane as a CO_2_ absorbent [109]. PLA membranes with a higher degree of crystallinity have been reported to be more permeable than PLA membranes with amorphous characteristics [141].

**Oil–water separation**

The separation of oil and water is a significant research topic with applications in the treatment of oily wastewater and the recovery of oil spills. Therefore, developing effective oil–water separation tools is of great importance to address these environmental issues. The selective adsorption of oil phase using materials with hydrophobic properties has provoked increased attention. Porous PLA membranes are anticipated to become efficient adsorption materials for oil–water separation. The addition of various fillers to the polymer matrix improves the adsorptive characteristics of porous polymeric materials. Li et al. created a new material by incorporating zeolite imidazole framework as a metal–organic framework (MOF) into a PLA-based matrix to obtain a more efficient membrane for oil–water separation [142]. The obtained material containing 1 wt% zeolite imidazole framework exhibited greater oil wettability and surface area, as well as the best oil–water separation performance.

Polymeric membranes exhibit a hydrophobic character. Su et al. prepared a superhydrophobic PLA membrane using the NIPS method [143]. Superhydrophobic membranes have special properties such as antifouling, self-cleaning, anti-icing, and can also be used to remove oil from oily wastewater.

**Medical applications and drug release control**

Due to the fact that PLA and PHB monomers are extracted from nontoxic bio-sources, their polymers have found extensive applications in the biomedical industry and in the field of drug delivery [15,49,144]. PLA or PLA blends can be used for drug release control for various medical agents such as contraceptives, vaccines, anesthetics, proteins, and peptides [49].

**Food packaging**

Because PLA is a biodegradable polymer, its use in food packaging has unlimited applications as a “greener” alternative to petroleum-based polymers [94,145]. Moreover, due to its antimicrobial and antifungal characteristics, PLA film represents a promising alternative for enhancing food safety by preventing the growth of microorganisms and bacteria [146,147,148]. The replacement of traditional food packaging plastics with biodegradable polymers is currently booming due to the reduction in greenhouse gas emissions and the development of sustainable materials. PLA-based membranes have several advantages, including mechanical properties, biocompatibility, gloss, transparency, tactile feel, and heat resistance [149].

**Food industry**

Membranes are widely used in the food industry, particularly in filtration. Some membranes are used for the separation and detection of food additives and the extraction of active ingredients. One of the methods employed is electrospinning technology [150].

Due to the large number of methyl groups in PLA molecules, their hydrophobicity is increased. Incorporating nanoparticles or blending with other polymers enhances water flux and improves performance in separation processes [151].

Recently, do Nascimento et al. published a review on innovative trends in the use of membranes in the food sector [152]. The efficiency of membranes in this sector depends on several factors, including their composition and various operational parameters. The use of anti-fouling strategies implies the modification of membranes to improve their performance. Although numerous methods are employed for membrane modification in industrial applications, challenges remain in this field. The processes generally used for membranes include microfiltration, ultrafiltration, nanofiltration, reverse osmosis, forward osmosis, electrodialysis, and membrane distillation. Each method has its own advantages and disadvantages. Membrane modification is employed to enhance their functions, such as membrane selectivity, flux, and efficiency. These methods can be categorized as chemical or physical. Physical methods, such as coating or blending, alter surface characteristics by applying a thin layer of different materials. Chemical methods include polymer functionalization, plasma treatment, or graft polymerization. Modified membranes are used in various food industry sectors, including oil, proteins, bioactive compounds, pectin, sugar, and fruit juice, among others [152]. Dibdiakova et al. used membranes for the separation and purification of proteins from chicken byproduct hydrolysate [153].

Membrane technology has recently been explored for the recovery of biologically active substances, such as caffeic acid, rosmarinic acid, and luteolin, from ethanol extracts of lavender residues [154]. The membrane exhibited good mechanical properties, indicating potential scalability, as it does not compromise the biological activity of concentrated compounds.

## 9. Conclusions

Membranes prepared from polylactic acid (PLA) and poly(3-hydroxybutyrate) (PHB) as bio-sourced and biodegradable polymers have been extensively studied in scientific literature. These types of membranes are a promising alternative to conventional petroleum-based membranes. Numerous studies have been conducted on the production of PLA and PHB from renewable biomass sources, including first- and second-generation biomass. PLA is a fully biodegradable polymer synthesized from lactic acid through either chemical or enzymatic processes. One of the most investigated issues in this topic is the separation of lactic acid from biomass.

PHAs are biodegradable aliphatic polyesters having a structure derived from 3-hydroxyalkanoic acid. PHB was the first discovered PHA. It is a short-chain linear homopolymer consisting of repeating units of 3-hydroxyalkanoate. PHB can be produced either synthetically or through fermentation using bacteria from various sources, including the genera *Alcaligenes*, *Bacillus*, and *Pseudomonas*.

The production of membranes from biobased polymers has received great interest in recent years. New types of biopolymers have been developed with improved characteristics, mainly regarding their mechanical and thermal resistance. Some innovative procedures, such as self-assembly, blending, surface grafting, or coating, have been applied to achieve this. Three main methodologies were developed to produce membranes from bio-based polymers: spinning, phase inversion, and 3D printing. Membranes produced from bio-sourced and biodegradable polymers have been successfully employed in numerous applications, including wastewater treatment, oil–water separation, gas separation, biomedical applications, drug delivery and release, and food packaging.

However, the high cost of these types of membranes remains one of the critical issues to be improved from a commercial perspective. Further research is still needed to identify low-cost substrates and suitable microorganisms that can enhance the manufacturing productivity of both PLA and PHB.

## Figures and Tables

**Table 1:** Characteristics of PLA and PHB.

Characteristics	PLA	PHB
Physico-chemical and mechanical		
Structure	Linear aliphatic polyester (levorotatory (L-), dextrorotatory (D-), meso (combination of L- and D-)) [40,41]	Microbial polyester, composed of 3-hydroxybutyrate monomers [38]
Functional groups	Ester (−COO−) linkages [42]	Methyl (–CH_3_) and ester (–COOR) groups [38]
Density	1.21–1.25 g/cm^3^ [43,44] 1.24–1.25 g/cm^3^ [45]	1.23–1.25 g/cm^3^ [44] 1.25 g/cm^3^ [43,46]
Crystallinity	37% [25,44,47]	50–60% [38] 60% [43,44,46]
Tensile strength	21–60 MPa [42,43,44] 45–70 MPa [25] 32–68 MPa [45]	20–40 MPa [38,48] 31 MPa [45] 40 MPa [46,47] 43 MPa [44]
Elongation at break	<10% [42] 2.5–6% [43,44]	6% [46] 7% [45] 5–10% [38]
Thermal		
Melting temperature (Tm)	150–162 °C [42,43,44] 160 °C [49] 170–183 °C [25] 173–178 °C [47] 130–180 °C [45,50]	160–180 °C [39] 168–182 °C [42] 177 °C [43,46] 175 °C [47] 165–175 °C [38] 171–182 °C [44]
Glass transition temperature (Tg)	45–60 °C [42,43,44] 60 °C [51] 55–65 °C [25,49] 60–65 °C [47] 50–80 °C [50] 60–80 °C [45]	2 °C [43,46,47] 2–15 °C [44] 5–9 °C [38] 15.0–5.0 °C [42]
Thermal degradation	200 °C [42,52] 230–260 °C [50] 215 °C [45]	180 °C [53] 220 and 290 °C [52]
Barrier		
Oxygen permeability	15.0–25.0 mL mm/m^2^ day atm [43] 1.94–2.30 m^3^ m/m^2^ s Pa [45]	2.0–10.0 mL mm/m^2^ day atm [43]
Water vapor permeability	5.0–7.0 g mm/m^2^ day [43] 1.2–2.2 kg m/m^2^ s Pa [45]	1.0–5.0 g mm/m^2^ day [43]
Other		
Biodegradability	Hydrolitic, enzymatic [43,45]	Microbial [46,54]
Hydrophilicity	Hydrophobic, water contact angle 70–80° [42,55]	Hydrophobic, water contact angle of 80–105° [56]
Solubility	Soluble in chloroform, methylene chloride, acetonitrile, 1,1,2-trichloroethane and dichloroacetic acid, dioxane [49,57] Insoluble in alcohols, water, linear hydrocarbons [57]	Soluble in chloroform, dichloromethane and chlorinated hydrocarbons [56,58] Insoluble in water, alcohols, organic solvents [59]
pH stability	Stable in neutral and acidic pH, degrades in alkaline pH [60]	Stable in neutral and more resistant in acidic pH, rapid hydrolysis in alkaline pH [61,62]

**Table 2:** Comparison of the characteristics of PLA and PHB.

Property	PLA	PHB
Origin	Bio-based (corn, sugarcane, wheat, cassava, and maize) and lignocellulosic biomass	Bio-based (carbon sources)
Production	Sugar extraction, lactic acid fermentation, polymerization, processing	Carbon source preparation, fermentation, PBB accumulation, extraction, purification and drying
Biodegradability	Biodegradable and compostable	Completely biodegradable
Degradation time	Months, years in natural environments	Weeks to months in soil or water
Degradation mechanism	Hydrolytic cleavage of ester to lactic acid	Hydrolytic and enzymatic cleavage to 3-hydroxybutyric acid
Environmental impact	Low, slower degradation	Very low, rapid degradation
Transparency	Transparent	Opaque
Thermal properties	Low thermal resistance	Higher than PLA, better heat resistance
Processability	Easy to process	Difficult to process (hard and brittle thermoplastic)
Mechanical properties	High tensile strengths	Good mechanical properties, comparable with polypropylene
Application	Packaging, agriculture, medical, textiles, fibers, etc.	Packaging, biomedical, textiles, agriculture, etc.
Cost	Lower production costs, widely available	Higher production cost, less commercially available

**Table 3:** A critical evaluation of the main characteristics of membrane preparation methods from bio-based polymers.

Method	Spinning	Phase Inversion	Three-Dimensional Printing
Basic Principle	Uses mechanical spinning or electrostatic forces to draw polymer membranes	Polymer is dissolved in a solvent and cast; then phase separation is applied	Shapes membranes layer-by-layer using a digital model.
Suitability for Biodegradable Polymers	High. Appropriate for porous structures. Solvent selection and degradation conditions needs optimization	Moderate to High. PLA is processable. Solvent/non-solvent systems must be compatible with biodegradability	Moderate. Limited by polymer melt or toner rheology.
Structural Control	Medium to High. Membrane or fiber diameter and pore size controlled by parameters like voltage, flow rate, and distance	Moderate. Can control morphology and porosity via solvent choice. Less precise control on pore architecture	Very High. Great potential for mimicking biological structures. User-defined macro/microporous architectures possible
Scalability and Cost	High scalability; Moderate costs-equipment cost is reasonable, but low throughput in electrospinning	Very high scalability; Low to moderate costs; well-established in industrial membrane production.	Low to Moderate scalability; High costs; slow printing speed, costly equipment and materials
Environmental Impact	Moderate to High. Depends on solvent system; Biodegradable polymers help reduce waste.	Variable. Use of toxic solvents is a concern; greener alternatives need development.	Low. Low material waste; often uses thermoplastic biodegradable polymers like PLA.
Application Suitability	Tissue scaffold, high-surface-area filtration membranes.	Water filtration, gas separation, controlled drug delivery	Customized implants, tissue engineering scaffolds, membranes requiring specific shapes
Challenges	Low throughput; uniformity and reproducibility issues.	Environmental and health concerns with solvents	Limited range of printable biodegradable polymers; slow production rate; expensive

## Data Availability

Not applicable.

## Abbreviations

The following abbreviations are used in this manuscript:

PLA	Polylactic acid
PHB	Poly(3-hydroxybutyrate)
PHAs	Polyhydroxyalkanoates
PVC	Poly(vinyl chloride)
PVDF	Polyvinylidene fluoride
PVDF HFP	Poly(vinylidene fluoride-co-hexafluoropropylene)
PHV	Polyhydroxyvalerate
PCL	Polycaprolactone
CA	Cellulose acetate
CTA	Cellulose triacetate
LAB	Lactic acid bacteria
PDLA	Poly(D-lactic acid)
PLLA	Poly(L-lactic acid)
PDLLA	Poly(DL-lactic acid)
DW	Dry weight
DMF	Dimethylformamide
DMSO	Dimethyl sulfoxide
TEP	Triethylene phosphate
ML	Methyl lactate
PHA	Polyhydroxyalkanoate
NIPS	Non-solvent-induced phase separation
TIPS	Thermally induced phase inversion
FDM	Fused deposition modeling
FTIR	Fourier-Transform Infrared Spectroscopy
SEM	Scanning Electron Microscopy
XRD	X-ray diffraction
TGA	Thermogravimetric analysis
DSC	Differential Scanning Calorimetry
sc-PLA	Stereocomplex-type polylactide
scl-PHA	Short-chain-length polyhydroxyalkanoate
mcl-PHA	Medium-chain-length polyhydroxyalkanoate
lcl-PHA	Long-chain-length polyhydroxyalkanoate
L-LDH	L-lactate dehydrogenase
D-LDH	D-lactate dehydrogenase
CALB	*Candida antarctica* lipase B
CRL	*Candida rugosa* lipase
LA	Lactic acid
C/N	Carbon-to-nitrogen
CO_2_	Carbon dioxide
Tm	Melting temperature
Tg	Glass transition temperature
PEO	Polyethyleneoxide
TEGDA	Triethylene glycol diacetate
ATBC	Acetyl tributyl citrate
SLS	Selective laser sintering
CHAp	Carbonated hydroxyapatite
ABS	Acrylonitrile butadiene styrene
HAp	Hydroxyapatite
PBS	Poly(butylene succinate
CNWs	Cellulose nanowhisker
MOF	Metal–organic framework
PIMs	Polymer inclusion membranes
GO	Graphene oxide
MF	Microfiltration
UF	Ultrafiltration
NF	Nanofiltration
RO	Reverse osmosis

## References

[1] Kolya H., Kang C.-W. (2023). Next-Generation Water Treatment: Exploring the Potential of Biopolymer-Based Nanocomposites in Adsorption and Membrane Filtration. Polymers.

[2] Senila M., Neag E., Tanaselia C., Senila L. (2023). Removal of Cesium and Strontium Ions from Aqueous Solutions by Thermally Treated Natural Zeolite. Materials.

[3] Dharupaneedi S.P., Nataraj S.K., Nadagouda M., Reddy K.R., Shukla S.S., Aminabhavi T.M. (2019). Membrane-based separation of potential emerging pollutants. Sep. Purif. Technol..

[4] Chai W.S., Cheun J.Y., Kumar P.S., Mubashir M., Majeed Z., Banat F., Ho S.-H., Show P.L. (2021). A review on conventional and novel materials towards heavy metal adsorption in wastewater treatment application. J. Clean. Prod..

[5] Senila M. (2025). Polymer Inclusion Membranes (PIMs) for Metal Separation—Toward Environmentally Friendly Production and Applications. Polymers.

[6] European Bioplastics Bioplastics Market Development Update 2024. https://www.european-bioplastics.org/market/.

[7] Rogovina S., Zhorina L., Gatin A., Prut E., Kuznetsova O., Yakhina A., Olkhov A., Samoylov N., Grishin M., Iordanskii A. (2020). Biodegradable Polylactide-Poly(3-Hydroxybutyrate) Compositions Obtained via Blending under Shear Deformations and Electrospinning: Characterization and Environmental Application. Polymers.

[8] D’Amico D.A., Iglesias Montes M.L., Manfredi L.B., Cyras V.P. (2016). Fully bio-based and biodegradable polylactic acid/poly(3-hydroxybutirate) blends: Use of a common plasticizer as performance improvement strategy. Polym. Test..

[9] Senila L., Kovacs E., Resz M.A., Senila M., Becze A., Roman C. (2024). Life Cycle Assessment (LCA) of Bioplastics Production from Lignocellulosic Waste (Study Case: PLA and PHB). Polymers.

[10] Saeidlou S., Huneault M.A., Li H., Park C.B. (2012). Poly(lactic acid) crystallization. Prog. Polym. Sci..

[11] Senila L., Gal E., Kovacs E., Cadar O., Dan M., Senila M., Roman C. (2023). Poly(3-hydroxybutyrate) Production from Lignocellulosic Wastes Using Bacillus megaterium ATCC 14581. Polymers.

[12] Butt F.I., Muhammad N., Hamid A., Moniruzzaman M., Sharif F. (2018). Recent progress in the utilization of biosynthesized polyhydroxyalkanoates for biomedical applications–Review. Int. J. Biol. Macromol..

[13] da Silva L.P., Kundu S.C., Reis R.L., Correlo V.M. (2020). Electric Phenomenon: A Disregarded Tool in Tissue Engineering and Regenerative Medicine. Trends Biotechnol..

[14] Cheng H., Yang X., Che X., Yang M., Zhai G. (2018). Biomedical application and controlled drug release of electrospun fibrous materials. Mater. Sci. Eng. C Mater. Biol. Appl..

[15] Tyler B., Gullotti D., Mangraviti A., Utsuki T., Brem H. (2016). Polylactic acid (PLA) controlled delivery carriers for biomedical applications. Adv. Drug Deliv. Rev..

[16] Bartczak Z., Galeski A., Kowalczuk M., Sobota M., Malinowski R. (2013). Tough blends of poly(lactide) and amorphous poly([R,S]-3-hydroxy butyrate)–morphology and properties. Eur. Polym. J..

[17] Rosli N.A., Ahmad I., Anuar F.H., Abdullah I. (2018). The contribution of eco-friendly bio-based blends on enhancing the thermal stability and biodegradability of Poly(lactic acid). J. Clean. Prod..

[18] Keawsupsak K., Jaiyu A., Pannoi J., Somwongsa P., Wanthausk N., Sueprasita P., Eamchotchawalit C. (2014). Poly (lactic acid)/biodegradable polymer blend for the preparation of flat-sheet membrane. J. Teknol..

[19] Findrik Balogova A., Hudak R., Toth T., Schnitzer M., Feranc J., Bakos D., Zivcak J. (2018). Determination of geometrical and viscoelastic properties of PLA/PHB samples made by additive manufacturing for urethral substitution. J. Biotechnol..

[20] Pavan F.A., Junqueira T.L., Watanabe M.D.B., Bonomi A., Quines L.K., Schmidell W., de Aragao G.M.F. (2019). Economic analysis of polyhydroxybutyrate production by Cupriavidus necator using different routes for product recovery. Biochem. Eng. J..

[21] Okolie O., Kumar A., Edwards C., Lawton L.A., Oke A., McDonald S., Thakur V.K., Njuguna J. (2023). Bio-Based Sustainable Polymers and Materials: From Processing to Biodegradation. J. Compos. Sci..

[22] Clarivate. https://www.webofscience.com/.

[23] Kim J.F., Thi H.Y.N., Török B. (2025). Biodegradable Synthetic Polymers. Encyclopedia of Green Chemistry.

[24] Lestido-Cardama A., Barbosa-Pereira L., Sendon R., Bustos J., Paseiro Losada P., Rodriguez Bernaldo de Quiros A. (2025). Chemical safety and risk assessment of bio-based and/or biodegradable polymers for food contact: A review. Food Res. Int..

[25] Khouri N.G., Bahú J.O., Blanco-Llamero C., Severino P., Concha V.O.C., Souto E.B. (2024). Polylactic acid (PLA): Properties, synthesis, and biomedical applications—A review of the literature. J. Mol. Struct..

[26] Rahmayetty, Whulanza Y., Sukirno, Rahman S.F., Suyono E.A., Yohda M., Gozan M. (2018). Use of Candida rugosa lipase as a biocatalyst for L-lactide ring-opening polymerization and polylactic acid production. Biocatal. Agric. Biotechnol..

[27] de Albuquerque T.L., Marques Júnior J.E., de Queiroz L.P., Ricardo A.D.S., Rocha M.V.P. (2021). Polylactic acid production from biotechnological routes: A review. Int. J. Biol. Macromol..

[28] Marques Junior J.E., de Queiroz L.P., de Albuquerque T.L., de Souza Zampieri D., Melo V.M.M., Rocha M.V.P. (2024). Lactic acid production from cashew apple bagasse, an agro-industrial waste, and its application in the enzymatic synthesis of polylactic acid. Biocatal. Agric. Biotechnol..

[29] Mou L., Li J., Lu Y., Li G., Li J. (2025). Polylactic acid: A future universal biobased polymer with multifunctional performance—From monomer synthesis, and processing to applications: A review. J. Hazard. Mater. Adv..

[30] Tan C., Tao F., Xu P. (2022). Direct carbon capture for the production of high-performance biodegradable plastics by cyanobacterial cell factories. Green Chem..

[31] Otitoju T.A., Kim C.-H., Ryu M., Park J., Kim T.-K., Yoo Y., Park H., Lee J.-H., Cho Y.H. (2024). Exploring green solvents for the sustainable fabrication of bio-based polylactic acid membranes using nonsolvent-induced phase separation. J. Clean. Prod..

[32] Yashavanth P.R., Das M., Maiti S.K. (2021). Recent progress and challenges in cyanobacterial autotrophic production of polyhydroxybutyrate (PHB), a bioplastic. J. Environ. Chem. Eng..

[33] Lin Y.-C., Ng I.S. (2025). Biofabrication of polyhydroxybutyrate (PHB) in engineered Cupriavidus necator H16 from waste molasses. J. Taiwan Inst. Chem. Eng..

[34] Kranert L., Kok R., Neumann A.S., Kienle A., Duvigneau S. (2025). Estimation of PHA concentrations from cell density data in Cupriavidus necator. Appl. Microbiol. Biotechnol..

[35] Fink P., Menzel C., Kwon J.H., Forchhammer K. (2025). A novel recombinant PHB production platform in filamentous cyanobacteria avoiding nitrogen starvation while preserving cell viability. Microb. Cell Fact..

[36] Russo G., Scocca P., Gelosia M., Fabbrizi G., Giannoni T., Urbani S., Esposto S., Nicolini A. (2024). Poly(3-hydroxybutyrate) production for food packaging from biomass derived carbohydrates by cupriavidus necator DSM 545. Enzym. Microb. Technol..

[37] Li Y., Wang Y., Wang R., Yan X., Wang J., Wang X., Chen S., Bai F., He Q., Yang S. (2022). Metabolic engineering ofZymomonas mobilisfor continuous co-production of bioethanol and poly-3-hydroxybutyrate (PHB). Green Chem..

[38] McAdam B., Brennan Fournet M., McDonald P., Mojicevic M. (2020). Production of Polyhydroxybutyrate (PHB) and Factors Impacting Its Chemical and Mechanical Characteristics. Polymers.

[39] Tang X., Thankappan S.K., Lee P., Fard S.E., Harmon M.D., Tran K., Yu X., Kumbar S.G., Laurencin C.T., Deng M. (2014). Chapter 21—Polymeric Biomaterials in Tissue Engineering and Regenerative Medicine. Natural and Synthetic Biomedical Polymers.

[40] Ranakoti L., Gangil B., Mishra S.K., Singh T., Sharma S., Ilyas R.A., El-Khatib S. (2022). Critical Review on Polylactic Acid: Properties, Structure, Processing, Biocomposites, and Nanocomposites. Materials.

[41] Sin L.T., Rahmat A.R., Rahman W.A.W.A., Sin L.T., Rahmat A.R., Rahman W.A.W.A. (2013). 4—Chemical Properties of Poly(lactic Acid). Polylactic Acid.

[42] Farah S., Anderson D.G., Langer R. (2016). Physical and mechanical properties of PLA, and their functions in widespread applications —A comprehensive review. Adv. Drug Deliv. Rev..

[43] Naser A.Z., Deiab I., Darras B.M. (2021). Poly(lactic acid) (PLA) and polyhydroxyalkanoates (PHAs), green alternatives to petroleum-based plastics: A review. RSC Adv..

[44] Roohi, Zaheer M.R., Kuddus M. (2017). PHB (poly-β-hydroxybutyrate) and its enzymatic degradation. Polym. Adv. Technol..

[45] Domenek S., Ducruet V. (2016). Characteristics and Applications of PLA. Biodegradable and Biobased Polymers for Environmental and Biomedical Applications.

[46] Rajan K.P., Thomas S.P., Gopanna A., Chavali M., Martínez L.M.T., Kharissova O.V., Kharisov B.I. (2017). Polyhydroxybutyrate (PHB): A Standout Biopolymer for Environmental Sustainability. Handbook of Ecomaterials.

[47] Kariduraganavar M.Y., Kittur A.A., Kamble R.R., Kumbar S.G., Laurencin C.T., Deng M. (2014). Chapter 1—Polymer Synthesis and Processing. Natural and Synthetic Biomedical Polymers.

[48] Zainuddin M.Z., Abu Bakar A.A., Adam A.N., Abdullah S.M., Tamchek N., Alauddin M.S., Mahat M.M., Wiwatcharagoses N., Alforidi A., Ghazali M.I. (2023). Mechanical and Structural Properties of Polyhydroxybutyrate as Additive in Blend Material in Additive Manufacturing for Medical Applications. Polymers.

[49] Ebrahimi F., Ramezani Dana H. (2021). Poly lactic acid (PLA) polymers: From properties to biomedical applications. Int. J. Polym. Mater. Polym. Biomater..

[50] Avérous L., Ebnesajjad S. (2013). 9—Synthesis, Properties, Environmental and Biomedical Applications of Polylactic Acid. Handbook of Biopolymers and Biodegradable Plastics.

[51] Bergström J.S., Hayman D. (2016). An Overview of Mechanical Properties and Material Modeling of Polylactide (PLA) for Medical Applications. Ann. Biomed. Eng..

[52] Kervran M., Vagner C., Cochez M., Ponçot M., Saeb M.R., Vahabi H. (2022). Thermal degradation of polylactic acid (PLA)/polyhydroxybutyrate (PHB) blends: A systematic review. Polym. Degrad. Stab..

[53] Hong S.-G., Hsu H.-W., Ye M.-T. (2013). Thermal properties and applications of low molecular weight polyhydroxybutyrate. J. Therm. Anal. Calorim..

[54] Lee J., Park H.J., Moon M., Lee J.-S., Min K. (2021). Recent progress and challenges in microbial polyhydroxybutyrate (PHB) production from CO_2_ as a sustainable feedstock: A state-of-the-art review. Bioresour. Technol..

[55] Luque-Agudo V., Hierro-Oliva M., Gallardo-Moreno A.M., González-Martín M.L. (2021). Effect of plasma treatment on the surface properties of polylactic acid films. Polym. Test..

[56] Liu Y., Li D., Ran X., Nie W., Semeniuk I., Koretska N. (2025). Synthesis, Structure, and Properties of Polyhydroxybutyrate Derived from Azotobacter Vinelandii N-15. ChemistryOpen.

[57] Casalini T., Rossi F., Castrovinci A., Perale G. (2019). A Perspective on Polylactic Acid-Based Polymers Use for Nanoparticles Synthesis and Applications. Front. Bioeng. Biotechnol..

[58] Lawley M.D., Boon D., Stein L.Y., Sauvageau D. (2022). Switchable Solvents for the Reversible Dissolution of Poly(3-hydroxybutyrate). ACS Sustain. Chem. Eng..

[59] Chaber P., Kwiecień M., Zięba M., Sobota M., Adamus G. (2017). The heterogeneous selective reduction of PHB as a useful method for preparation of oligodiols and surface modification. RSC Adv..

[60] Vaid R., Yildirim E., Pasquinelli M.A., King M.W. (2021). Hydrolytic Degradation of Polylactic Acid Fibers as a Function of pH and Exposure Time. Molecules.

[61] Chen L.X.L., Yu J. (2005). Abiotic Hydrolysis of Poly[(R)-3-hydroxybutyrate] in Acidic and Alkaline Media. Macromol. Symp..

[62] Tapadiya A., Vasanthan N. (2017). Crystallization and alkaline hydrolysis of poly(3-hydroxybutyrate) films probed by thermal analysis and infrared spectroscopy. Int. J. Biol. Macromol..

[63] Qi Y., Ma H.-L., Du Z.-H., Yang B., Wu J., Wang R., Zhang X.-Q. (2019). Hydrophilic and Antibacterial Modification of Poly(lactic acid) Films by γ-ray Irradiation. ACS Omega.

[64] Wang J., Huang J., Liu S. (2024). The production, recovery, and valorization of polyhydroxybutyrate (PHB) based on circular bioeconomy. Biotechnol. Adv..

[65] Zhuikov V.A., Akoulina E.A., Chesnokova D.V., Wenhao Y., Makhina T.K., Demyanova I.V., Zhuikova Y.V., Voinova V.V., Belishev N.V., Surmenev R.A. (2020). The Growth of 3T3 Fibroblasts on PHB, PLA and PHB/PLA Blend Films at Different Stages of Their Biodegradation In Vitro. Polymers.

[66] Attallah O.A., Mojicevic M., Garcia E.L., Azeem M., Chen Y., Asmawi S., Brenan Fournet M. (2021). Macro and Micro Routes to High Performance Bioplastics: Bioplastic Biodegradability and Mechanical and Barrier Properties. Polymers.

[67] Goonoo N., Bhaw-Luximon A., Passanha P., Esteves S., Schönherr H., Jhurry D. (2017). Biomineralization potential and cellular response of PHB and PHBV blends with natural anionic polysaccharides. Mater. Sci. Eng. C.

[68] Akdoğan E., Şirin H.T., Şahal G., Deniz Z., Kaya A., Serdaroğlu D.Ç. (2023). Accelerating the environmental biodegradation of poly-3-hydroxybutyrate (PHB) via plasma surface treatment. Bioresour. Technol. Rep..

[69] Arrieta M., Samper M., Aldas M., López J. (2017). On the Use of PLA-PHB Blends for Sustainable Food Packaging Applications. Materials.

[70] More N., Avhad M., Utekar S., More A. (2022). Polylactic acid (PLA) membrane—Significance, synthesis, and applications: A review. Polym. Bull..

[71] Vatanpour V., Dehqan A., Paziresh S., Zinadini S., Zinatizadeh A.A., Koyuncu I. (2022). Polylactic acid in the fabrication of separation membranes: A review. Sep. Purif. Technol..

[72] Xix-Rodriguez C., Varguez-Catzim P., Alonzo-García A., Rodriguez-Fuentes N., Vázquez-Torres H., González-Diaz A., Aguilar-Vega M., González-Díaz M.O. (2021). Amphiphilic poly(lactic acid) membranes with low fouling and enhanced hemodiafiltration. Sep. Purif. Technol..

[73] Ehsani M., Kalugin D., Doan H., Lohi A., Abdelrasoul A. (2022). Bio-Sourced and Biodegradable Membranes. Appl. Sci..

[74] Granados-Hernandez M.V., Serrano-Bello J., Montesinos J.J., Alvarez-Gayosso C., Medina-Velazquez L.A., Alvarez-Fregoso O., Alvarez-Perez M.A. (2018). In vitro and in vivo biological characterization of poly(lactic acid) fiber scaffolds synthesized by air jet spinning. J. Biomed. Mater. Res. Part B Appl. Biomater..

[75] Abdal-hay A., Sheikh F.A., Lim J.K. (2013). Air jet spinning of hydroxyapatite/poly(lactic acid) hybrid nanocomposite membrane mats for bone tissue engineering. Colloids Surf. B Biointerfaces.

[76] An Tran N.H., Brünig H., Hinüber C., Heinrich G. (2013). Melt Spinning of Biodegradable Nanofibrillary Structures from Poly(lactic acid) and Poly(vinyl alcohol) Blends. Macromol. Mater. Eng..

[77] Fryczkowski R., Fryczkowska B., Biniaś W., Janicki J. (2013). Morphology of fibrous composites of PLA and PVDF. Compos. Sci. Technol..

[78] Zhu Y., Gu X., Dong Z., Wang B., Jin X., Chen Y., Cui M., Wang R., Zhang X. (2023). Regulation of polylactic acid using irradiation and preparation of PLA–SiO_2_–ZnO melt-blown nonwovens for antibacterial and air filtration. RSC Adv..

[79] Guo Y., Wu M., Ye X., Wei S., Huang L., Guo H. (2025). High-Efficiency and Low-Resistance Melt-Blown/Electrospun PLA Composites for Air Filtration. Polymers.

[80] Xue J., Wu T., Dai Y., Xia Y. (2019). Electrospinning and Electrospun Nanofibers: Methods, Materials, and Applications. Chem. Rev..

[81] Zhao J., Liu X., Pu X., Shen Z., Xu W., Yang J. (2024). Preparation Method and Application of Porous Poly(lactic acid) Membranes: A Review. Polymers.

[82] Zhang W., Liu H., Yan L., Mei X., Hou Z. (2023). Combining emulsion electrospinning with surface functionalization to fabricate multistructural PLA/CS@ZIF-8 nanofiber membranes toward pH-responsive dual drug delivery. Int. J. Biol. Macromol..

[83] Zhao Y., Ming J., Cai S., Wang X., Ning X. (2023). One-step fabrication of polylactic acid (PLA) nanofibrous membranes with spider-web-like structure for high-efficiency PM0.3 capture. J. Hazard. Mater..

[84] Kahrs C., Schwellenbach J. (2020). Membrane formation via non-solvent induced phase separation using sustainable solvents: A comparative study. Polymer.

[85] Jung J.T., Kim J.F., Wang H.H., di Nicolo E., Drioli E., Lee Y.M. (2016). Understanding the non-solvent induced phase separation (NIPS) effect during the fabrication of microporous PVDF membranes via thermally induced phase separation (TIPS). J. Membr. Sci..

[86] Cano-Vicent A., Tambuwala M.M., Hassan S.S., Barh D., Aljabali A.A.A., Birkett M., Arjunan A., Serrano-Aroca Á. (2021). Fused deposition modelling: Current status, methodology, applications and future prospects. Addit. Manuf..

[87] Zhang H.Y., Jiang H.B., Ryu J.-H., Kang H., Kim K.-M., Kwon J.-S. (2019). Comparing Properties of Variable Pore-Sized 3D-Printed PLA Membrane with Conventional PLA Membrane for Guided Bone/Tissue Regeneration. Materials.

[88] Choi W.J., Hwang K.S., Kwon H.J., Lee C., Kim C.H., Kim T.H., Heo S.W., Kim J.-H., Lee J.-Y. (2020). Rapid development of dual porous poly(lactic acid) foam using fused deposition modeling (FDM) 3D printing for medical scaffold application. Mater. Sci. Eng. C.

[89] Yehia H.M., Hamada A., Sebaey T.A., Abd-Elaziem W. (2024). Selective Laser Sintering of Polymers: Process Parameters, Machine Learning Approaches, and Future Directions. J. Manuf. Mater. Process..

[90] Duan B., Wang M., Zhou W.Y., Cheung W.L., Li Z.Y., Lu W.W. (2010). Three-dimensional nanocomposite scaffolds fabricated via selective laser sintering for bone tissue engineering. Acta Biomater..

[91] Rosenzweig D., Carelli E., Steffen T., Jarzem P., Haglund L. (2015). 3D-Printed ABS and PLA Scaffolds for Cartilage and Nucleus Pulposus Tissue Regeneration. Int. J. Mol. Sci..

[92] Heo D.N., Castro N.J., Lee S.-J., Noh H., Zhu W., Zhang L.G. (2017). Enhanced bone tissue regeneration using a 3D printed microstructure incorporated with a hybrid nano hydrogel. Nanoscale.

[93] Bikiaris N.D., Koumentakou I., Samiotaki C., Meimaroglou D., Varytimidou D., Karatza A., Kalantzis Z., Roussou M., Bikiaris R.D., Papageorgiou G.Z. (2023). Recent Advances in the Investigation of Poly(lactic acid) (PLA) Nanocomposites: Incorporation of Various Nanofillers and their Properties and Applications. Polymers.

[94] Ramezani Dana H., Ebrahimi F. (2022). Synthesis, properties, and applications of polylactic acid-based polymers. Polym. Eng. Sci..

[95] Ferreira P.S., Ribeiro S.M., Pontes R., Nunes J. (2024). Production methods and applications of bioactive polylactic acid: A review. Environ. Chem. Lett..

[96] Luo N., Stewart M.J., Hirt D.E., Husson S.M., Schwark D.W. (2004). Surface modification of ethylene-co-acrylic acid copolymer films: Addition of amide groups by covalently bonded amino acid intermediates. J. Appl. Polym. Sci..

[97] Wang S., Cui W., Bei J. (2005). Bulk and surface modifications of polylactide. Anal. Bioanal. Chem..

[98] Pan J., Wang Y., Qin S., Zhang B., Luo Y. (2005). Grafting reaction of poly(D,L)lactic acid with maleic anhydride and hexanediamine to introduce more reactive groups in its bulk. J. Biomed. Mater. Res. Part B Appl. Biomater..

[99] Tejada-Oliveros R., Fiori S., Gomez-Caturla J., Lascano D., Montanes N., Quiles-Carrillo L., Garcia-Sanoguera D. (2022). Development and Characterization of Polylactide Blends with Improved Toughness by Reactive Extrusion with Lactic Acid Oligomers. Polymers.

[100] Lim L.T., Auras R., Rubino M. (2008). Processing technologies for poly(lactic acid). Prog. Polym. Sci..

[101] de Kort G.W., Bouvrie L.H.C., Rastogi S., Wilsens C.H.R.M. (2019). Thermoplastic PLA-LCP Composites: A Route toward Sustainable, Reprocessable, and Recyclable Reinforced Materials. ACS Sustain. Chem. Eng..

[102] Darie-Niță R.N., Irimia A., Doroftei F., Stefan L.M., Iwanczuk A., Trusz A. (2023). Bioactive and Physico-Chemical Assessment of Innovative Poly(lactic acid)-Based Biocomposites Containing Sage, Coconut Oil, and Modified Nanoclay. Int. J. Mol. Sci..

[103] Backes E.H., de Nóbile Pires L., Selistre-de-Araujo H.S., Costa L.C., Passador F.R., Pessan L.A. (2020). Development and characterization of printable PLA/β-TCP bioactive composites for bone tissue applications. J. Appl. Polym. Sci..

[104] Mukaffa H., Asrofi M., Sujito, Asnawi, Hermawan Y., Sumarji, Qoryah R.D.H., Sapuan S.M., Ilyas R.A., Atiqah A. (2022). Effect of alkali treatment of piper betle fiber on tensile properties as biocomposite based polylactic acid: Solvent cast-film method. Mater. Today Proc..

[105] Stoll L., Rech R., Flôres S.H., Nachtigall S.M.B., de Oliveira Rios A. (2019). Poly(acid lactic) films with carotenoids extracts: Release study and effect on sunflower oil preservation. Food Chem..

[106] Qasim U., Osman A.I., Al-Muhtaseb A.H., Farrell C., Al-Abri M., Ali M., Vo D.-V.N., Jamil F., Rooney D.W. (2020). Renewable cellulosic nanocomposites for food packaging to avoid fossil fuel plastic pollution: A review. Environ. Chem. Lett..

[107] Abdulkareem A., Kasak P., Nassr M.G., Mahmoud A.A., Al-Ruweidi M.K.A.A., Mohamoud K.J., Hussein M.K., Popelka A. (2021). Surface Modification of Poly(lactic acid) Film via Cold Plasma Assisted Grafting of Fumaric and Ascorbic Acid. Polymers.

[108] Nakagawa M., Teraoka F., Fujimoto S., Hamada Y., Kibayashi H., Takahashi J. (2006). Improvement of cell adhesion on poly(L-lactide) by atmospheric plasma treatment. J. Biomed. Mater. Res. Part A.

[109] Akbarzadeh E., Shockravi A., Vatanpour V. (2021). High performance compatible thiazole-based polymeric blend cellulose acetate membrane as selective CO_2_ absorbent and molecular sieve. Carbohydr. Polym..

[110] Filippova E.O., Zhuravleva A.D., Gorbunova E.A., Ivanova N.M. (2020). The influence of implantation of plasma-modified polylactic acid films on the structure of the cornea. AIP Conf. Proc..

[111] Huang Y., Wang Y., Li Y., Luo C., Yang C., Shi W., Li L. (2020). Covalent Immobilization of Polypeptides on Polylactic Acid Films and Their Application to Fresh Beef Preservation. J. Agric. Food Chem..

[112] Quirk R.A., Davies M.C., Tendler S.J.B., Shakesheff K.M. (2000). Surface Engineering of Poly(lactic acid) by Entrapment of Modifying Species. Macromolecules.

[113] Zhu H., Ji J., Lin R., Gao C., Feng L., Shen J. (2002). Surface engineering of poly(dl-lactic acid) by entrapment of alginate-amino acid derivatives for promotion of chondrogenesis. Biomaterials.

[114] Wang C., Mao L., Yao J., Zhu H. (2023). Improving the active food packaging function of poly(lactic acid) film coated by poly(vinyl alcohol) based on proanthocyanidin functionalized layered clay. LWT.

[115] Danafar H., Rostamizadeh K., Davaran S., Hamidi M. (2016). Drug-conjugated PLA–PEG–PLA copolymers: A novel approach for controlled delivery of hydrophilic drugs by micelle formation. Pharm. Dev. Technol..

[116] Hazarika D., Kumar A., Katiyar V. (2022). Structural evolution of in situ polymerized poly(L-lactic acid) nanocomposite for smart textile application. Sci. Rep..

[117] DeStefano V., Khan S., Tabada A. (2020). Applications of PLA in modern medicine. Eng. Regen..

[118] Nazeer M.A., Onder O.C., Sevgili I., Yilgor E., Kavakli I.H., Yilgor I. (2020). 3D printed poly(lactic acid) scaffolds modified with chitosan and hydroxyapatite for bone repair applications. Mater. Today Commun..

[119] Saniei H., Mousavi S. (2020). Surface modification of PLA 3D-printed implants by electrospinning with enhanced bioactivity and cell affinity. Polymer.

[120] Imani F., Karimi-Soflou R., Shabani I., Karkhaneh A. (2021). PLA electrospun nanofibers modified with polypyrrole-grafted gelatin as bioactive electroconductive scaffold. Polymer.

[121] Canales D.A., Reyes F., Saavedra M., Peponi L., Leonés A., Palza H., Boccaccini A.R., Grünewald A., Zapata P.A. (2022). Electrospun fibers of poly (lactic acid) containing bioactive glass and magnesium oxide nanoparticles for bone tissue regeneration. Int. J. Biol. Macromol..

[122] Arrieta M.P., López J., Hernández A., Rayón E. (2014). Ternary PLA–PHB–Limonene blends intended for biodegradable food packaging applications. Eur. Polym. J..

[123] Ma P., Spoelstra A.B., Schmit P., Lemstra P.J. (2013). Toughening of poly (lactic acid) by poly (β-hydroxybutyrate-co-β-hydroxyvalerate) with high β-hydroxyvalerate content. Eur. Polym. J..

[124] Jasek V., Fucik J., Ivanova L., Vesely D., Figalla S., Mravcova L., Sedlacek P., Krajcovic J., Prikryl R. (2022). High-Pressure Depolymerization of Poly(lactic acid) (PLA) and Poly(3-hydroxybutyrate) (PHB) Using Bio-Based Solvents: A Way to Produce Alkyl Esters Which Can Be Modified to Polymerizable Monomers. Polymers.

[125] Khajavian M., Salehi E., Vatanpour V. (2020). Chitosan/polyvinyl alcohol thin membrane adsorbents modified with zeolitic imidazolate framework (ZIF-8) nanostructures: Batch adsorption and optimization. Sep. Purif. Technol..

[126] Shokri E., Khanghahi B., Esmizadeh E., Etemadi H. (2021). Biopolymer-based adsorptive membrane for simultaneous removal of cationic and anionic heavy metals from water. Int. J. Environ. Sci. Technol..

[127] Zia Q., Tabassum M., Lu Z., Khawar M.T., Song J., Gong H., Meng J., Li Z., Li J. (2020). Porous poly(L-lactic acid)/chitosan nanofibres for copper ion adsorption. Carbohydr. Polym..

[128] Zia Q., Tabassum M., Meng J., Xin Z., Gong H., Li J. (2021). Polydopamine-assisted grafting of chitosan on porous poly (L-lactic acid) electrospun membranes for adsorption of heavy metal ions. Int. J. Biol. Macromol..

[129] Mohammad N., Atassi Y. (2020). TiO2/PLLA Electrospun Nanofibers Membranes for Efficient Removal of Methylene Blue Using Sunlight. J. Polym. Environ..

[130] Modolon H.B., Teixeira L.B., Mazur L.P., Santos P.H., Camani P.H., Mei L.H.I., Wermuth T.B., Montedo O.R.K., Zimmermann M.V.G., Arcaro S. (2025). Electrospun adsorbent membrane of PLA containing chitosan for toxic metal ions removal from aqueous solution: Effect of chitosan incorporation. Int. J. Biol. Macromol..

[131] Kian L.K., Jawaid M., Nasef M.M., Fouad H., Karim Z. (2021). Poly(lactic acid)/poly(butylene succinate) dual-layer membranes with cellulose nanowhisker for heavy metal ion separation. Int. J. Biol. Macromol..

[132] Prazeres Mazur L., Reis Ferreira R., Felix da Silva Barbosa R., Henrique Santos P., Barcelos da Costa T., Gurgel Adeodato Vieira M., da Silva A., dos Santos Rosa D., Helena Innocentini Mei L. (2024). Development of novel biopolymer membranes by electrospinning as potential adsorbents for toxic metal ions removal from aqueous solution. J. Mol. Liq..

[133] Mokoena L.S., Mofokeng J.P. (2024). Preparation of poly(lactic acid) (PLA)/poly(3-hydroxybutyrate-co-3-hydroxyvalerate) (PHBV)/graphene oxide (GO) polymeric composites for the selective removal of lead ions (Pb(II)) in water. Polym. Compos..

[134] Nigiz F.U., Tan B., Bektaş T.E., Karakoca B. (2025). A comparative study on removal of boron via pervaporation and vacuum membrane distillation using zirconium metal–organic framework-loaded poly(lactic acid) membrane. Appl. Water Sci..

[135] Bożejewicz D., Witt K., Kaczorowska M.A. (2023). Influence of the type of polymer and plasticizer on the properties and efficiency of membranes containing acetylacetone carrier for the removal of Cd(II) ions from aqueous solutions. Desalination Water Treat..

[136] Hammadi M.H., Kerakra S., Bey S., Sellami F., Djermoune A., Habi A. (2024). Advancements in Cr(VI) Removal from Aqueous Solution Using PLA/PBAT/GO/Cloisite 30b Hybrid Nanocomposite Polymer Inclusion Membranes. Water Air Soil Pollut..

[137] Matei E., Covaliu C.I., Coman G., Negroiu M., Rapa M., Predescu A.-M., Berbecaru A.-C., Predescu C. (2021). Nonwoven Bio-Based Membranes for Removal of Micropollutants from Aqueous Water. Mater. Plast..

[138] Cairone S., Hegab H.M., Khalil H., Nassar L., Wadi V.S., Naddeo V., Hasan S.W. (2023). Novel eco-friendly polylactic acid nanocomposite integrated membrane system for sustainable wastewater treatment: Performance evaluation and antifouling analysis. Sci. Total Environ..

[139] Morales-Jimenez M., Palacio D.A., Palencia M., Melendrez M.F., Rivas B.L. (2023). Bio-Based Polymeric Membranes: Development and Environmental Applications. Membranes.

[140] Lehermeier H.J., Dorgan J.R., Way J.D. (2001). Gas permeation properties of poly(lactic acid). J. Membr. Sci..

[141] Iulianelli A., Algieri C., Donato L., Garofalo A., Galiano F., Bagnato G., Basile A., Figoli A. (2017). New PEEK-WC and PLA membranes for H2 separation. Int. J. Hydrogen Energy.

[142] Li Y., Lin Z., Wang X., Duan Z., Lu P., Li S., Ji D., Wang Z., Li G., Yu D. (2021). High-hydrophobic ZIF-8@PLA composite aerogel and application for oil-water separation. Sep. Purif. Technol..

[143] Su Y., Zhao Y., Zheng W., Yu H., Liu Y., Xu L. (2020). Asymmetric Sc-PLA Membrane with Multi-scale Microstructures: Wettability, Antifouling, and Oil-Water Separation. ACS Appl. Mater. Interfaces.

[144] Jain A., Kunduru K.R., Basu A., Mizrahi B., Domb A.J., Khan W. (2016). Injectable formulations of poly(lactic acid) and its copolymers in clinical use. Adv. Drug Deliv. Rev..

[145] Yanat M., Schroën K. (2021). Preparation methods and applications of chitosan nanoparticles; with an outlook toward reinforcement of biodegradable packaging. React. Funct. Polym..

[146] Motelica L., Ficai D., Ficai A., Oprea O.C., Kaya D.A., Andronescu E. (2020). Biodegradable Antimicrobial Food Packaging: Trends and Perspectives. Foods.

[147] Fahmy H.M., Salah Eldin R.E., Abu Serea E.S., Gomaa N.M., AboElmagd G.M., Salem S.A., Elsayed Z.A., Edrees A., Shams-Eldin E., Shalan A.E. (2020). Advances in nanotechnology and antibacterial properties of biodegradable food packaging materials. RSC Adv..

[148] Priyadarshi R., Rhim J.-W. (2020). Chitosan-based biodegradable functional films for food packaging applications. Innov. Food Sci. Emerg. Technol..

[149] Yang W., Hao J., Wu Y., Zhou Y., Mi L., Hu Y. (2025). Electrostatic spinning construction of GA/HP-β-CD functionalized COS/PLA composite membrane for cherry tomato preservation applications. Food Packag. Shelf Life.

[150] Dong H., Tong L., Cheng M., Hou S. (2024). Utilizing electrospun molecularly imprinted membranes for food industry: Opportunities and challenges. Food Chem..

[151] Cai J., Han Z., Sun Y., Chen H., Li H., Wang R., Ji Y., He B. (2025). A novel, green method for the preparation of biodegradable PLA membranes without pore-forming agent. J. Water Process. Eng..

[152] do Nascimento N.N., Paraíso C.M., Molina L.C.A., Dzyazko Y.S., Bergamasco R., Vieira A.M.S. (2024). Innovative Trends in Modified Membranes: A Mini Review of Applications and Challenges in the Food Sector. Membranes.

[153] Dibdiakova J., Matic J., Wubshet S.G., Uhl W., Manamperuma L.D., Rusten B., Vik E.A. (2024). Membrane Separation of Chicken Byproduct Hydrolysate for Up-Concentration of Bioactive Peptides. Membranes.

[154] Stoyanova Y., Lazarova-Zdravkova N., Peshev D. (2025). Is Membrane Filtration Applicable for the Recovery of Biologically Active Substances from Spent Lavender?. Membranes.

